# Macrophage inflammatory and regenerative response periodicity is programmed by cell cycle and chromatin state

**DOI:** 10.1016/j.molcel.2022.11.017

**Published:** 2022-12-14

**Authors:** Bence Daniel, Julia A. Belk, Stefanie L. Meier, Andy Y. Chen, Katalin Sandor, Zsolt Czimmerer, Zsofia Varga, Krisztian Bene, Frank A. Buquicchio, Yanyan Qi, Hugo Kitano, Joshua R. Wheeler, Deshka S. Foster, Michael Januszyk, Michael T. Longaker, Howard Y. Chang, Ansuman T. Satpathy

**Affiliations:** 1 Department of Pathology, Stanford University, Stanford, CA 94305, USA; 2 Department of Computer Science, Stanford University, Stanford, CA 94305, USA; 3 Center for Personal Dynamic Regulomes, Stanford University, Stanford, CA 94305, USA; 4 Howard Hughes Medical Institute, Stanford University, Stanford, CA 94305, USA; 5 Hagey Laboratory for Pediatric Regenerative Medicine, Division of Plastic and Reconstructive Surgery, Stanford University, Stanford, CA 94305, USA; 6 Department of Surgery, Stanford University School of Medicine, Stanford CA 94305, USA; 7 Institute for Stem Cell Biology and Regenerative Medicine, Stanford University School of Medicine, Stanford, CA 94305, USA; 8 Department of Bioengineering, Stanford University, Stanford, CA 94305, USA; 9 Parker Institute for Cancer Immunotherapy, San Francisco, CA 94129, USA; 10 Program in Immunology, Stanford University, Stanford, CA, USA; 11 Department of Biochemistry and Molecular Biology, University of Debrecen, Debrecen, H-4032, Hungary; 12 Institute of Genetics, Biological Research Centre, Eötvös Loránd Research Network, Szeged, H-6726, Hungary; 13 Gladstone-UCSF Institute of Genomic Immunology, San Francisco, CA 94158, USA; 14 Lead contact

## Abstract

Cell cycle (CC) facilitates cell division via robust, cyclical gene expression. Protective immunity requires the expansion of pathogen-responsive cell types, but whether CC confers unique gene expression programs that direct the subsequent immunological response remains unclear. Here we demonstrate that single macrophages (MFs) adopt different plasticity states in CC, which leads to heterogeneous cytokine-induced polarization programs. Specifically, MF plasticity to interferon gamma (IFNG) is substantially reduced during S-G2/M, while interleukin 4 (IL-4) induces S-G2/M-biased gene expression, which can be mediated by CC-biased enhancers. Additionally, IL-4 polarization shifts the CC-phase distribution of MFs towards the G2/M-phase, providing a subpopulation-specific mechanism for IL-4-induced, dampened IFNG responsiveness. Finally, we demonstrate CC-dependent MF responses in multiple murine and human disease settings *in vivo*, including Th2-driven airway inflammation and pulmonary fibrosis, where MFs express an S-G2/M-biased tissue remodeling gene program. Therefore, MF inflammatory and regenerative responses are gated by CC in a cyclical, phase-dependent manner.

## eTOC Blurb

Daniel et al. report a single-cell chromatin atlas of macrophage polarization and discover cyclical macrophage plasticity states in cell cycle. They show that macrophage polarization is a cell cycle-dependent immunological process and uncover a cell cycle intrinsic tissue remodeling gene program in proliferating macrophages, detectable in tissue regeneration and fibrosis.

## Graphical Abstract

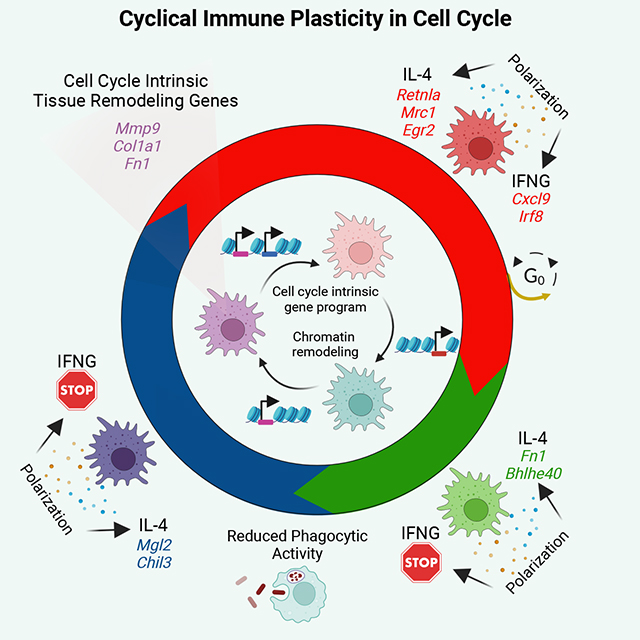

## Introduction

Cellular plasticity describes the phenotypic flexibility and responsiveness of a cell type in a changing microenvironment, a feature that is critical to adapt to environmental challenges. How plasticity is established in a population of cells is a key question in many biological systems, and certain cell types possess the ability to adopt more nuanced phenotypic traits in response to stressors and can also revert from this state; thus, being more plastic. In the immune system, this cellular feature is particularly important in patrolling, long-lived innate immune cell types.

Macrophages (MF) are innate immune cells with remarkable plasticity. As resident cells of various organs, MFs adopt distinct phenotypes to maintain tissue integrity and resolve infections. MFs achieve this by adjusting their epigenetic and gene expression programs according to changes in the microenvironment, a phenomenon called MF polarization.^1–4^ In the lung, alveolar MFs respond to infections but also have an important regulatory role in surfactant metabolism.^4^ Similarly, Kupffer cells of the liver respond to pathogens but also metabolize toxic or carcinogenic compounds.^5^ The pleiotropic actions of MF subpopulations across tissues indicates the existence of diverse MF plasticity states, tuning their responses at the subpopulation level when they undergo phenotypic polarization upon environmental challenges. Indeed, single-cell studies have begun to reveal MF heterogeneity in multiple tissues and cancer that likely bear distinct features of plasticity.^6–8^ Importantly, a common property of MFs is their proliferative potential in tissues of residence, which can be induced by MF growth factors (e.g., macrophage colony-stimulating factor [M-CSF], and granulocyte-macrophage colony-stimulating factor [GM-CSF]) and the T-helper 2 (Th2)-type cytokine, interleukin 4 (IL-4), resulting in cell cycle (CC) entry.^9–13^ MF proliferation replenishes the tissue-resident pool in homeostatic and pathological conditions and has been linked to the resolving phase of inflammation and tissue regeneration.^13–17^ However, whether entry into CC influences MF plasticity or polarization capacity has not been determined.

To uncover the phenotypic plasticity of MFs, bone marrow-derived MF (BMDM) models of classical (stimulation with interferon gamma (IFNG) or lipopolysaccharide (LPS) – referred to as M1 MFs) and alternative (stimulation with IL-4 or IL-13 – referred to as M2 MFs) polarization have been used as the gold-standard approach to understand the molecular principles of MF responses *in vitro*.^18–21^ This model is generally representative of circulating monocyte-derived macrophage responses and can be useful to mimic robust MF responses that can also occur *in vivo* in complex tissue environments that establish a spectrum of MF polarization states. For example, *bona fide* M1 MFs (*Nos2*^+^, *Il1b*^+^, *Tnf*^+^) are present during bacterial or viral infections, while M2 MFs (*Chil3*^+^, *Retnla*^+^, *Arg1*^+^) have been observed in wound healing, helminth infections, and allergic reactions.^18,19,21^ Therefore, this model can identify fundamental mechanisms of MF plasticity and may also translate to *in vivo* settings.

MF polarization has largely been studied at the population level, and our view of this process lacks sub-population level analyses.^22–30^ This apparent gap raises fundamental questions about MF plasticity at single-cell resolution: (1) what are the major determinants of MF plasticity across a population of cells? and (2) are there cell-intrinsic properties that influence MF plasticity to polarizing signals? Motivated by these questions, we sequenced ~30,000 bone marrow-derived MF transcriptomes (scRNA-seq) and *cis*-regulomes (scATAC-seq) to build a comprehensive (epi)genomic atlas of IFNG-induced (M1) and IL-4-induced (M2) macrophage polarization. We found heterogeneous MF polarization states that coincided with CC, and we show that CC influences MF plasticity during polarization. Interestingly, MFs lose their plasticity to IFNG in the S-G2/M-phases of CC, while IL-4 can induce a specific gene signature in S-G2/M, which can be perturbed by CRISPR editing of CC-biased enhancers. We find that CC reduces the formation of a chromatin imprint that defines a subpopulation of “memory” MFs after M2 polarization. Additionally, CC limits MF repolarization with IFNG from a M2 state. Finally, we discover a tissue remodeling gene signature in the S-G2/M-phases of CC that can also be detected in proliferating MFs during Th2-driven inflammation, muscle regeneration, and human idiopathic pulmonary fibrosis. Our work connects CC to MF immune responses, whereby inflammatory and regenerative MF responses are gated by CC in a cyclical, phase-dependent manner.

## Results

### Single-cell chromatin accessibility landscape of MF polarization.

**Figure 1.**
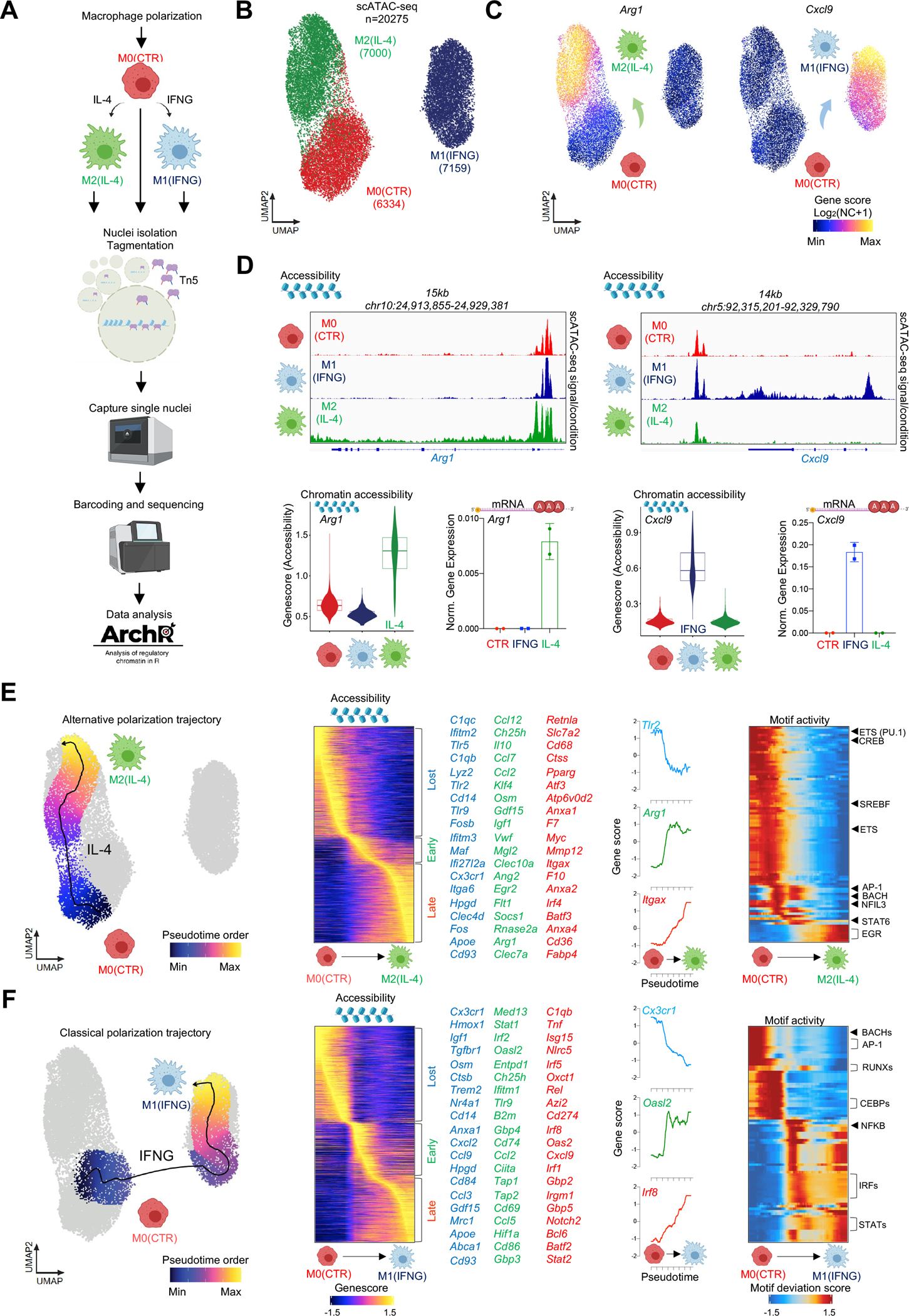
Heterogeneous cis-regulatory program of MF polarization. **(A)** Scheme of the experimental system. **(B)** UMAP projection of scATAC-seq results from polarized MFs. **(C)** UMAP projection of gene score values for *Arg1* and *Cxcl9*. **(D)** Aggregated scATAC-seq signal in the *Arg1* and *Cxcl9* loci (top). Gene score values and mRNA expression of *Arg1* and *Cxcl9* in CTR (red), IFNG (blue) and IL-4 (green) polarized MFs as determined by scATAC-seq and RT-qPCR (bottom). Data are represented as means ± SD. **(E)** UMAP projection of the alternative polarization trajectory (left). Heatmap of gene scores changing over the polarization trajectory. Genes that lose- (Lost - blue), gain early- (Early - green), or late (Late - red) accessibility are marked and a select set is displayed. *Tlr2*, *Arg1* and *Itgax* gene scores are shown over pseudotime. ChromVAR transcription factor motif deviation scores over pseudotime on the Alternative MF polarization trajectory. **(F)** Same as panel E; for the Classical MF polarization trajectory.

To understand MF heterogeneity at the chromatin level, we performed single-cell assay for transposase accessible chromatin using sequencing (scATAC-seq) in mouse bone marrow-derived resting (unstimulated; M0; CTR), classically-polarized (M1; IFNG), and alternatively-polarized MFs (M2; IL-4; Figure 1A). In total, we obtained high-quality scATAC-seq data from 20,275 single cells (Figure S1A and S1B). We performed dimensionality reduction using iterative latent semantic indexing (LSI) followed by UMAP visualization, which revealed a clear separation between M0, M1, and M2 MF chromatin states (Figure 1B). Known M2 (*Arg1*) and M1 (*Cxcl9*) polarization marker genes exhibited specific chromatin remodeling in the respective polarization states, correlating with bulk gene expression levels (Figure 1C and D). Transcription factor (TF) footprint analysis in M2 and M1 MFs showed strong footprints at known M2 (STAT6 and EGR) and M1 (IRF and STAT1) TF motifs, respectively, confirming our polarization model, and recapitulating previously described hallmarks of MF polarization (Figure S1C).^23,29,30^

We observed a continuum of MF polarization states which prompted us to assess polarization trajectories to describe phenotypic state transitions.^31^ We ordered single-cell MF chromatin states along a vector that describes the paths of the two main polarization trajectories on the UMAP. We reconstructed both M1 and M2 polarization trajectories by the nearest-neighbor approach, sequentially selecting MFs with similar chromatin states based on Euclidean distance.^31,32^ We observed “early” and “late” chromatin remodeling activities in the trajectories, such as early chromatin closure at repressed genes (e.g., M2 – *Tlr2* and *Cd14*; M1 – *Cx3cr1* and *Cd14*) and opening around “early” induced genes (e.g., M2 – *Arg1*, and *Egr2*; M1 – *Irf2* and *Stat1*).^23^ We also observed “late” chromatin remodeling activities that were induced at terminal stages of polarization (e.g., M2 – *Retnla* and *Pparg*; M1 – *Irf8* and *Cxcl9*; Figure 1E and F; Table S1 and S3). Chromatin remodeling dynamics of gene bodies followed mRNA expression from bulk, time course RNA-seq experiments in M2 MFs, suggesting that studying the transitional chromatin states of single MFs can recapitulate the cascade of gene regulatory events of MF polarization (Figure S1D and S1E).^23^ Motif accessibility analyses over the pseudotime trajectories linked TF motifs to the transitional open chromatin states of polarization (STAT6 and EGR2 TF motifs to M2 MFs, IRF and STAT1 TF motifs to M1 MFs; Figure 1E, F and S1F; Table S2 and S4). Our single-cell chromatin atlas describes the dynamic and heterogeneous chromatin states of M1 and M2 MFs and led us to investigate the major drivers of heterogeneity.

### MF heterogeneity coincides with cell cycle.

**Figure 2.**
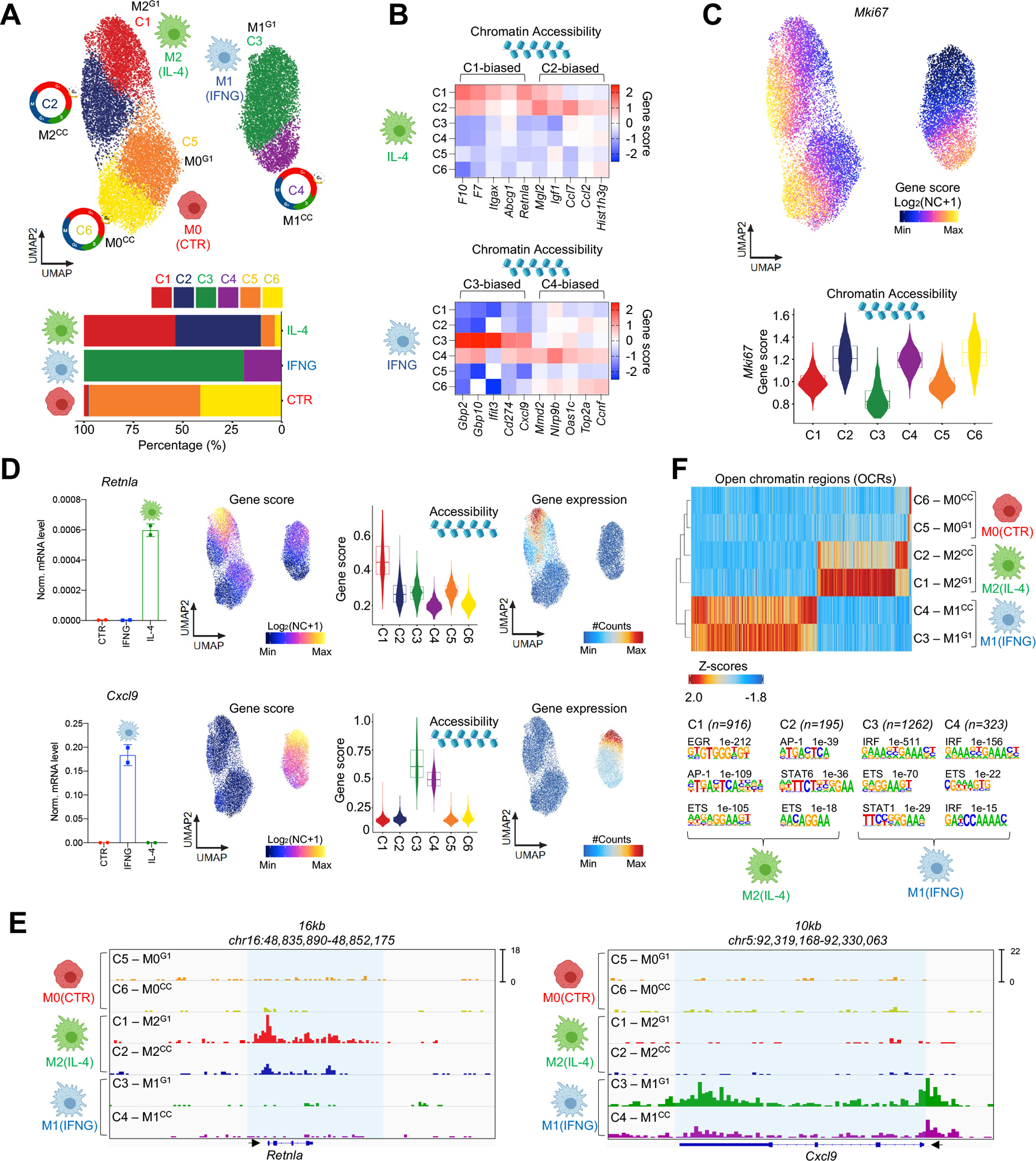
MF heterogeneity coincides with cell cycle markers. **(A)** scATAC UMAP of MF polarization colored by the 6 MF clusters. Percentage-wise distribution of the clusters across conditions (bottom). **(B)** Heatmap representation of marker gene scores in the polarizaed MF clusters. **(C)** UMAP of *Mki67* gene scores. Violin plot of *Mki67* gene scores in the clusters. **(D)** Bar graphs depict bulk mRNA levels of *Retnla* and *Cxcl9* (left). Data are represented as means ± SD. UMAPs and violin plots show gene score values (log_2_ normalized counts+1) (scATAC-seq) for the two genes (middle). UMAPs of gene integration scores (gene expression - scRNA-seq), # - normalized. **(E)** Genome browser snapshots of scATAC-seq signal intensities in the 6 clusters for *Retnla* and *Cxcl9*. **(F)** Peak score heatmap of differentially accessible cis-regulatory regions in the clusters. Homer *de novo* motif search results. Number of regions in each cluster and p-values for the enriched motifs are shown.

We identified two distinct chromatin state clusters in each polarization condition (M0 – C5 and C6; M2 – C1 and C2; M1 – C3 and C4; Figure 2A). In general, polarized MFs did not co-cluster with M0 MFs; however, approximately 10% of MFs from the IL-4 polarized condition exhibited a M0 phenotype (Figure 2A). Next, we identified the marker gene scores of each cluster (FDR≤0.01, Log_2_ fold change (FC)≥1.25; Table S5). We observed C1- (n=261) and C2-biased (n=113) gene scores (e.g., C1 – *Retnla* and *Abcg1*; C2 - *Mgl2*, and *Igf1*). Conversely, M1 MFs exhibited C3- (n=483) and C4-biased gene scores (n=317), including *bona fide* M1 marker genes (e.g., C3 - *Gbp2*, *and Cxcl9*; C4 - *Mmd2*, and *Oas1c*; Figure 2B). Importantly, gene score values of cell cycle (CC)-related genes were largely specific to one of the two clusters in each condition. Namely, *Hist1h3g*, *Top2a*, *Ccnf,* and *Mki67* exhibited high gene score values in C2 of M2, C4 of M1, and C6 of M0 MFs, compared to the other clusters, indicating that MFs are in CC (Figure 2B and 2C). For clarity, we annotated cycling MF clusters with the superscript CC (M0 C6 – M0^CC^, M1 C4 – M1^CC^, M2 C2 – M2^CC^), and non-cycling clusters that are likely detected in the G0/G1 phase with the superscript G1 (M0 C5 – M0^G1^, M1 C3 – M1^G1^, M2 C1 – M2^G1^).

We performed single-cell RNA-seq in M0, M1 and M2 MFs to link mRNA expression to chromatin changes and identified differentially expressed genes between M0 – M2 (Induced: 214, Repressed 147) and M0 – M1 MFs (Induced: 494, Repressed: 212; FDR≤0.01, Log_2_ FC≥0.25; Figure S2A and S2B; Table S6). Constrained integration of single-cell chromatin and transcriptomic profiles of polarization states generated a gene integration matrix with pseudo-scRNA-seq expression values for each MF in the scATAC-seq space, recapitulating cluster-specific chromatin accessibility profiles (Figure S2C).^32^ Most importantly, expression of CC genes was specific to CC clusters in each polarization state (M0^CC^, M1^CC^, and M2^CC^). Polarized MFs in CC exhibited reduced expression and accessibility for specific M1 (e.g., *Cxcl9* and *Gbp2*) and M2 genes (e.g., *Retnla* and *Egr2*; Figure 2D, E and S2C). These results show that MF heterogeneity coincides with CC, and suggests that MF plasticity may be influenced by CC.

### The open chromatin landscape of M1 and M2 MF polarization is constrained by cell cycle.

Next, we defined the open chromatin regions (OCRs) of polarized MF subsets. In M2 MFs, we identified 916 M2^G1^-biased and 195 M2^CC^-biased OCRs. In M1 MFs, 1,262 M1^G1^- and 323 M1^CC^-biased OCRs were detected (FDR≤0.01, Log_2_ FC≥1; Figure 2F). M1^CC^ and M2^CC^ MFs showed smaller OCR changes upon polarization, compared to non-cycling MFs; however, we also noted a smaller set of polarization-induced OCRs that were biased to the CC-clusters. Motif enrichment analyses at the M2-specific OCRs identified the EGR2 motif in M2^G1^, while the STAT6 motif showed enrichment in M2^CC^-specific OCRs. In M1 MFs, we detected the STAT1 motif exclusively in M1^G1^, while the IRF motif was present in both cluster-specific OCR sets but showed biased enrichment in M1^G1^, compared to M1^CC^ (top 3 hits, p-values: C3 – 1e^−511^ versus C4 – 1e^−156^; Figure 2F). At the single-cell level, we found heterogeneous chromatin accessibility states around these TF motifs, demonstrating smaller differences between the clusters (Figure S2D). Finally, to assess if cluster-specific OCRs are likely engaged in gene regulation and represent transcriptionally active enhancers, we projected bulk RNAPII ChIP-seq signal from M0, M1, and M2 polarized MFs onto these genomic positions and found robust, cytokine-induced RNAPII recruitment (Figure S2E). These results suggest that chromatin remodeling activities of polarizing MFs are altered in CC.

### Cell cycle limits the expression of two key transcription factors of MF polarization.

**Figure 3.**
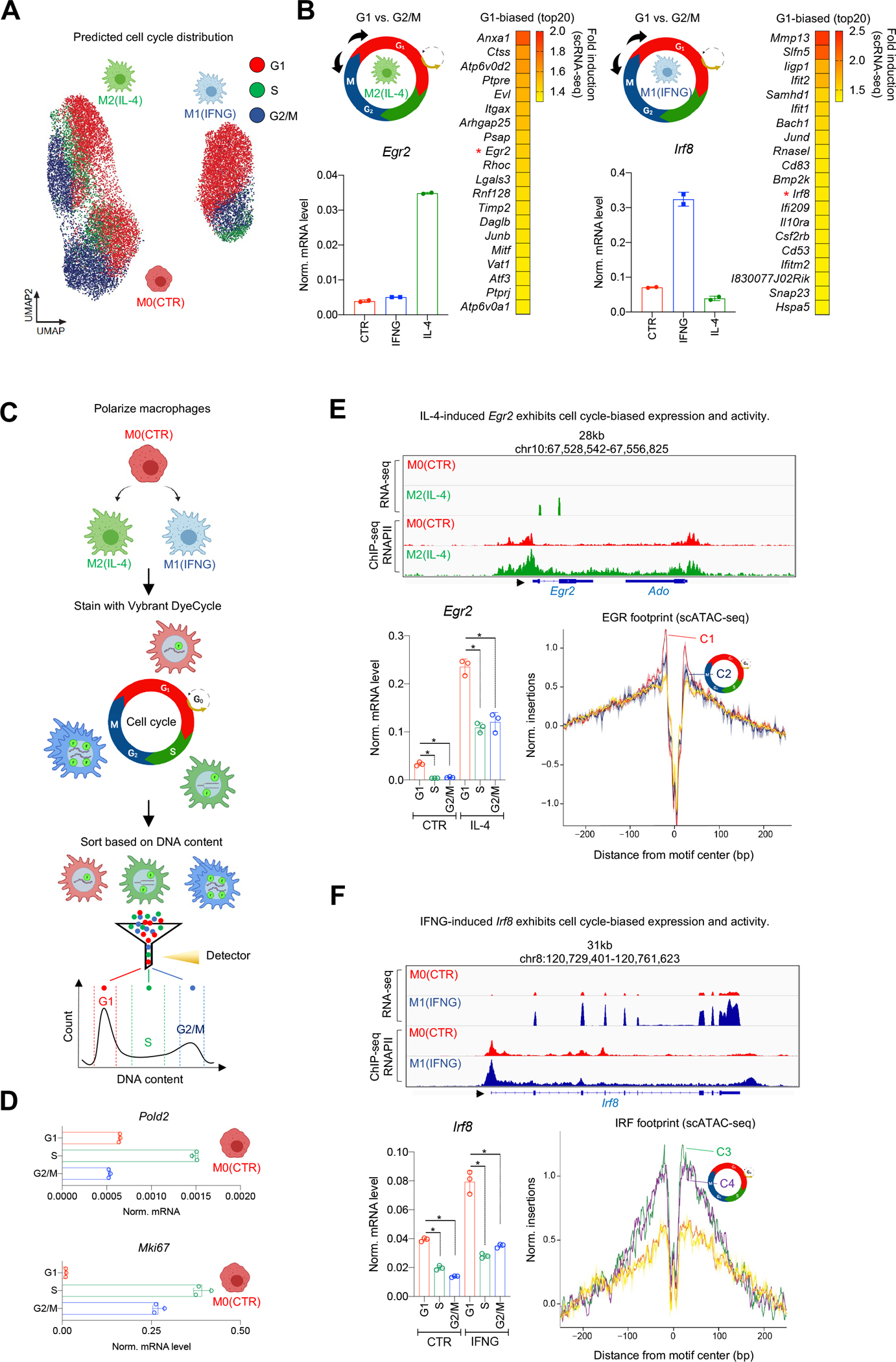
Cell cycle limits the expression of *Egr2* and *Irf8* during polarization. **(A)** UMAP of cell cycle scores in the polarized MF populations. **(B)** Differential gene expression analysis of G1 and G2/M predicted cells from the polarized states (scheme). Heatmap of genes exhibiting G1-biased expression in polarized MFs (right). *Egr2* and *Irf8* transcription factors are marked by red asterisks and their bulk expression level is validated by RT-qPCR. **(C)** Scheme of cell cycle sorting. **(D)** mRNA levels of *Pold2* and *Mki67* measured by RT-qPCR in cell cycle phase-sorted MFs. **(E)** Genome browser view of bulk RNA-seq and RNAPII ChIP-seq results in the *Egr2* locus in polarized MFs. mRNA levels of *Egr2* in cell cycle phases. Statistics: two tailed, unpaired t-test at p<0.05 (n=3). Data are represented as means ± SD. EGR transcription factor footprints in the 6 scATAC clusters. **(F)** Same as panel E; for *Irf8*.

To investigate whether CC alters MF plasticity, we took a predictive approach and used our gene integration matrix to assign specific CC stages (G1, S and G2/M) to each cell in the scATAC-seq space using a CC scoring algorithm (Figure 3A).^33^ This analysis showed that ~80% of MFs in M2 and more than 95% of cells in M0 and M1 were predicted to be in CC in the CC clusters (Figure S3A). Differential gene expression analyses (FDR≤0.01, FC≥1.3) between CC stages, using gene markers of M2 and M1 MFs that we previously defined (top 50 genes; Figure S2B, Table S6), predicted that polarization-induced gene expression is largely biased to the G1-phase (M1: 38 G1-biased genes; M2: 33 G1-biased genes; Figure 3B). Interestingly, some M2 genes showed S- (n=6; e.g., *Atpv0d2* and *Anxa1*) and G2/M-biased (n=11; e.g., *Mgl2* and *Gatm*) expression patterns, suggesting that CC might be more permissive for IL-4-triggered changes (Figure S3B). In M1 MFs, we found negligible number of genes with S- or G2/M-biased expression (S vs. G1 – n=2; S vs. G2/M – n=1; G2/M vs. S – n=2; G2/M vs. G1 – n=2), suggesting that CC reduced IFNG responsiveness (Figure 3B and S3B). Finally, we found reduced expression of polarization markers in CC, including the two driver TFs of M2 and M1 polarization, *Egr2* and *Irf8*, respectively (Figure 3B, Figure S3C and S3D).^30,34^

We quantified the CC distribution of M0 MFs by fluorescence-activated cell sorting (FACS) with a DNA labeling dye (Vybrant DyeCycle) and confirmed that MFs can be detected in different CC stages (~73% in G1, ~12% in S, and ~6% in G2/M), in agreement with our genomics data and a previous study (Figure 3C and S3E).^35^ We sorted F4/80^+^ M0 MFs from each CC phase and measured gene expression of phase-specific genes (S - *Pold2* and G2/M - *Mki67*) by real-time quantitative PCR (RT-qPCR), which showed that pure MF populations could be enriched from CC stages by this strategy (Figure 3D).^36^ Next, we measured the expression of *Egr2* and *Irf8* by RT-qPCR, which are readily induced by either IL-4 or IFNG, respectively, which validated their G1-biased expression, and was further supported by TF footprint analyses among cycling and non-cycling MFs from scATAC-seq data (Figure 3E and F). These results indicate that MF plasticity to polarization is reduced by CC.

### Cell cycle phase-dependent MF plasticity influences polarization potential.

**Figure 4.**
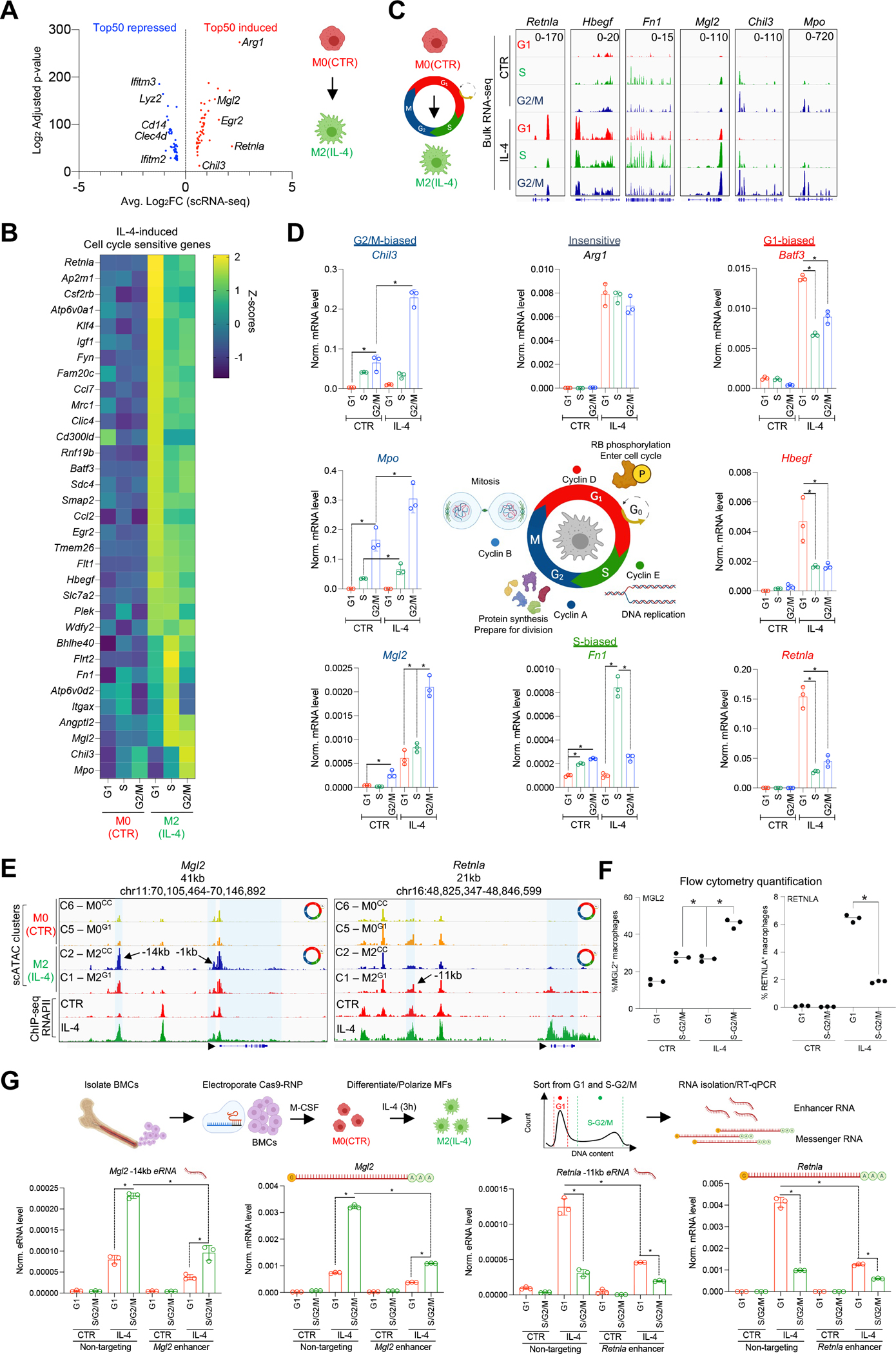
Cell cycle phase influences MF plasticity to polarization signals. **(A)** Volcano plot of the top 50 differentially expressed gene upon M2(IL-4) polarization determined by scRNA-seq. **(B)** Heatmap of cell cycle-phase sensitive, IL-4-induced genes determined by bulk RNA-seq. **(C)** Genome browser snapshots of genes that exhibit phase-biased expression. **(D)** Validation of cell cycle phase-biased gene expression by RT-qPCR. **(E)** Genome browser snapshots depict scATAC-seq and RNAPII ChIP-seq data in the *Mgl2* and *Retnla* loci in polarized MFs. **(F)** Quantification of MGL2 and RENTLA positive MFs in G1 and S-G2/M cell cycle-phases by flow cytometry. **(G)** Scheme of CRISPR perturbation experiments. RT-qPCR measurements of *Mgl2* and *Retnla* eRNAs and mRNA transcripts in the presence of a non-targeting and enhancer-targeting guide RNAs that target the indicated enhancers. Statistics throughout the figure: two tailed, unpaired t-test at p<0.05, n=3. Data are represented as means ± SD.

Next, we experimentally tested the effects of CC on MF plasticity. We performed bulk RNA-seq experiments in M0, M1, and M2 MFs sorted from CC-phases. To streamline the analysis, we used the top 50 polarization-induced and -repressed genes from scRNA-seq (Figure 4A and S4A, Table S6).^21^ First, using the bulk RNA-seq results, we defined CC-sensitive genes with differential expression profiles between any two CC-phases in each condition, yielding a total of 8,700 genes (Benjamini–Hochberg adjusted p≤0.001; FC≥1.3). Then, we overlapped this list with our top 50 induced and repressed marker genes of the two polarization models. We found that 74% of the M2 gene expression program was sensitive to CC (74/100 genes). Namely, 66% of the induced genes and 82% of the repressed genes exhibited CC-phase-dependent expression. Similarly, 76% of the core M1 polarization program was CC-sensitive (76/100 genes); 84% of the induced and 68% of the repressed genes displayed CC-phase-biased expression (Figure S4B).

Analysis of CC-phase-biased expression of IL-4-induced genes revealed G1- (48%, 24/50) and S-G2/M-biased gene expression (18%, 9/50). In both groups, we detected *bona fide* M2 MF marker genes (e.g., G1-biased: *Retnla*, *Mrc1*, *Egr2*, and S-G2/M-biased: *Fn1*, *Mgl2*, and *Chil3*; Figure 4B and C, Table S9). In contrast, 82% of the IFNG-induced genes (41/50 genes) exhibited G1-biased expression (e.g., *Cxcl9*, *Ifi44*, and *Irf8*), and only 3 genes showed S-G2/M-biased expression (*Ccl12*, *Apobec3* and *Pnp*; Figure S4C and D, Table S9). Among the 50 induced genes in the two polarization models, we found 17 IL-4- (e.g., *Arg1* and *Ptpre*) and 6 IFNG-induced (e.g., *Cxcl10* and *Irf1*), but CC-insensitive genes (Figure S4E). Repressed genes exhibited strong, phase-biased expression in M0 MFs in both polarization models. 62% of IL-4 repressed genes showed G1-biased expression (e.g., *Cd14* and *Clec4d*), and 25% displayed S-G2/M-biased expression (e.g., *Cx3cr1* and *Ifi27l2a*) in the M0 condition, while 44% of IFNG repressed genes exhibited G1-biased expression (e.g., *Ifngr1* and *C5ar1*), and 24% exhibited S-G2/M-biased expression, including genes that are required for DNA replication, in agreement with the finding that IFNG triggers CC arrest at the G1-S border in MFs (e.g., *Rps28* and *Gmnn*) (Figure S4F).^37^ Therefore, repression occurs by silencing phase-biased gene expression in the M0 state.

Importantly, CC inhibitors that arrest cells in G1- (Ribociclib) or G2-phases (Artesunate) further supported these results. Specifically, Ribociclib reduced *Mki67* expression and facilitated the expression of G1-biased genes in both polarization paradigms (IL-4 – *Retnla*; IFNG – *Ifit1*, *Irf8* and *Cxcl9*), while Artesunate induced the expression of *Mki67* and reduced the expression of these genes. In contrast, Ribociclib reduced, while Artesunate facilitated the expression of the IL-4-induced, G2/M-biased genes, such as *Mgl2* and *Chil3*. (Figure S4G). Since IL-4 can specifically induce gene expression in the S-G2M-phases, we focused on the M2 program, and validated these results by RT-qPCR (Figure 4D). Of these genes, we also validated the CC-phase-biased protein expression of MGL2, RETNLA, and MRC1 (G0/G1-biased) by FACS (Figure 4F and S4H and S4I). To test the functional relevance of these findings, we determined whether G1-biased expression of MRC1, a critical phagocytic receptor in MFs, was associated with reduced phagocytic activity in S-G2/M. We used two fluorescently-labeled bacterial particles derived from *Escherichia Coli* and *Staphylococcus Aureus* and confirmed that MFs exhibited reduced phagocytic activity towards these bacterial particles in S-G2/M, compared to G1 (Figure S4J). Collectively, these findings show that MF polarization and phagocytic activity is CC sensitive.

### Phase-biased enhancer activities regulate cell cycle stage-dependent *Mgl2* and *Retnla* expression.

We hypothesized that phase-biased enhancer activities might drive gene expression; therefore, we identified enhancers by projecting RNAPII ChIP-seq signal to OCRs that exhibited M1^G1^, M1^CC^, or M2^G1^, M2^CC^-biased accessibility, confirming that these OCRs selectively recruited RNAPII in the presence of the polarization signal that established them (Figure S2E). Next, we annotated these enhancers to the nearest genes with CC-biased expression from bulk RNA-seq, identifying 15 (e.g., *Mgl2* – G2-biased, *Retnla* – G0/G1-biased) and 74 (e.g., *Ctsc* – G0/G1-biased, *Apobec3* – G2/M-biased) CC-biased enhancers in M2 and M1 MFs, respectively (Figure S4K).

We focused on two major components of the M2-induced polarization program, *Mgl2* and *Retnla* (involved in the pathogenesis of multicellular parasite infections, allergic reactions, and asthma), as demonstrating either G2/M- or G1-biased IL-4-induced expression patterns and enhancer activities, respectively.^38–40^ First, we validated the CC-phase-biased expression of both genes (Figure 4D and F). Second, we measured enhancer RNA (eRNA – marker of enhancer activity) expression at their putative enhancers by RT-qPCR (Figure 4E).^41,42^ In the *Mgl2* locus, we detected G2/M-phase-dependent enhancer activity at the −1kb enhancer in M0 MFs, while the −14kb was silent. However, IL-4 readily induced eRNA production at both enhancers. The −1kb enhancer displayed weak IL-4-induced activity exclusively in the G2/M-phase, while the −14kb region showed strong induction in all phases, with higher G2/M-biased activity (Figure 4E and S4L). We also identified a candidate enhancer region (−11kb) in the *Retnla* locus and measured enhancer activity. Although this region did not show IL-4 induced accessibility, it was preferentially open in M0^G1^ and M2^G1^ MFs; however, we detected IL-4-induced RNAPII occupancy at this element, along with IL-4-induced eRNA production in a strongly G1-biased manner (Figure 4E and S4L).

To test the functional importance of these enhancers in CC-biased gene expression, we performed CRISPR-perturbations with single guide RNAs that target the STAT6 TF motifs in the distant *Mgl2* (−14kb) and *Retnla* (−11kb) enhancers, respectively. Targeting the *Mgl2*-associated enhancer reduced the S-G2/M-biased expression of the gene, as well as the eRNA transcript produced from the perturbed locus. Similarly, targeting the enhancer of *Retnla* led to reduced G1-biased gene and eRNA expression (Figure 4G). Therefore, these results confirmed that enhancers identified using our scATAC-seq analysis underlie CC phase-dependent gene expression.

### IL-4 priming imprints a memory chromatin signature in a subpopulation of MFs and is limited by cell cycle.

**Figure 5.**
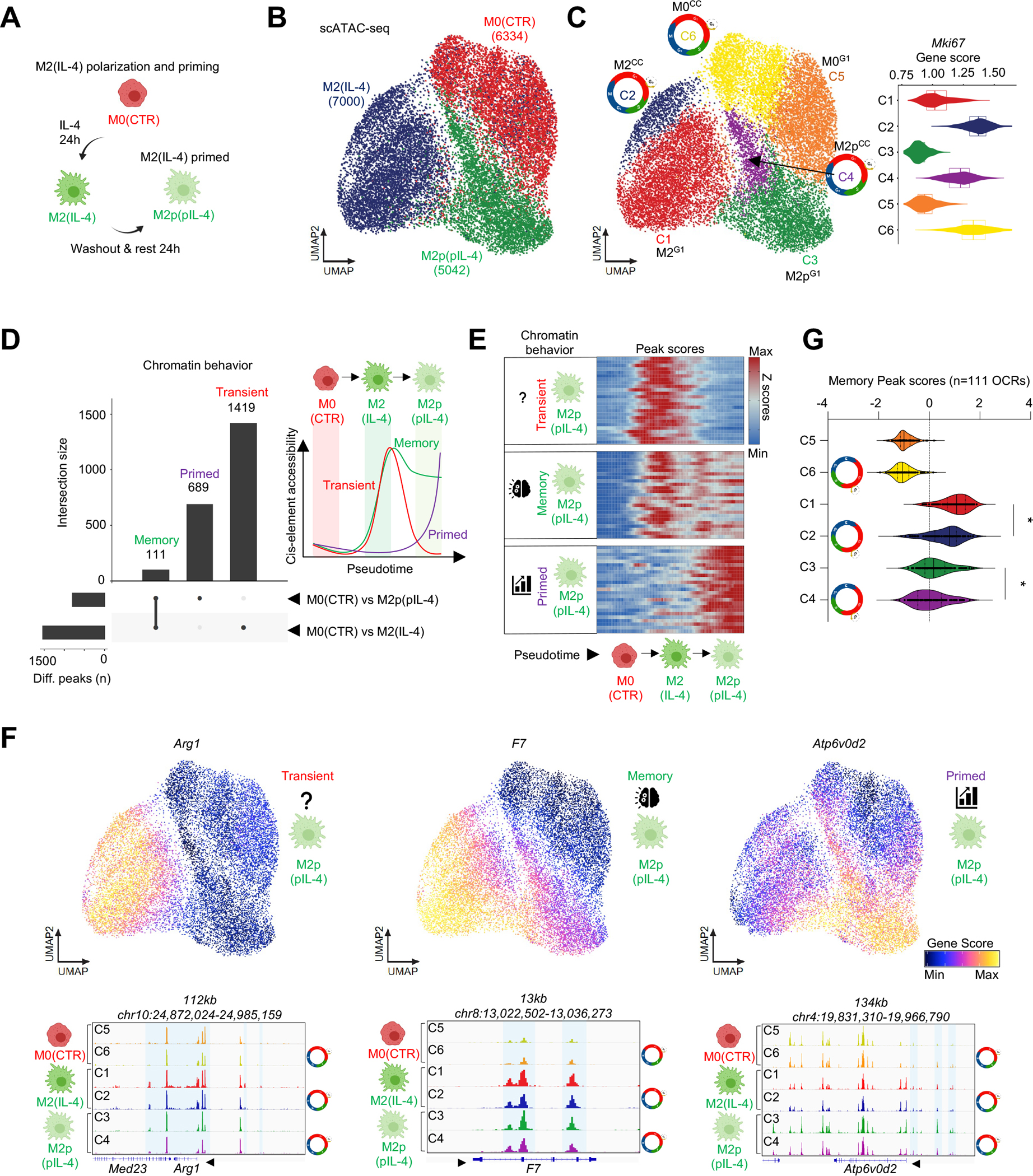
Cell cycle negatively affects the formation of memory in a MF subset at the chromatin level. **(A)** Scheme of the priming model. **(B)** scATAC-seq UMAP of M0(CTR), M2(IL-4) and primed M2p(pIL-4) MFs. **(C)** UMAP colored by the 6 clusters identified. Violin plot depicts the gene score values of *Mki67* in the 6 clusters. Cell cycle icons highlight cycling MF clusters. **(D)** Upset plot of the differentially accessible cis-elements in among the indicated conditions, and their overlap, yielding “memory”, “primed” and “transient” chromatin features. Scheme of revealed chromatin behaviors. **(E)** Heatmap of peak scores that exhibit chromatin behaviors from panel D over the indicated pseudotime trajectory. 25 peaks are shown **(F)** UMAPs depict gene score values for the indicated genes that display different chromatin behaviors. Genome browser views depict scATAC-seq signal in the indicated gene loci. **(G)** Violin plot depicts of the distribution of “Memory” peak scores (accessibility) across the clusters. Statistics: Wilcoxon Signed Rank Test between medians, p<0.0001.

Next, we asked whether CC also affects memory formation at the chromatin level in MFs. Since M2 polarization by IL-4 has been shown to reprogram MF responses to secondary stimuli, indicative of memory, we established a MF priming model and performed scATAC-seq (Figure 5A).^22,23,28^ UMAP projection of M0, M2, and M2p MFs (n=18,376 cells) suggested that IL-4-induced chromatin changes are largely transient; however, the majority (~95%) of MFs from the M2p condition remained distinct from M2 or M0 MFs in UMAP space (Figure 5B).

Clustering MF chromatin states resulted in 6 clusters (M0 – C5 and C6; M2 – C1 and C2; M2p – C3 and C4). Among these, C2 (M2^CC^), C4 (M2p^CC^) and C6 (M0^CC^) showed high *Mki67* accessibility; thus, we designated them as CC-clusters (Figure 5C). Differential OCR analysis revealed that IL-4-induced chromatin changes were largely transient, but cells in the primed state did display a subset of stable OCRs. Namely, of 1,530 IL-4-induced OCRs, 1,419 returned to steady state (“transient”), while 111 retained accessibility after IL-4-washout (“memory”; FDR≤0.01, Log_2_ FC≥1; Figure 5D). We also observed 689 OCRs with induced accessibility following IL-4-washout (“primed“; Figure 5D, Table S7). To establish a sequence of chromatin remodeling events in the priming model, we reconstructed a potential priming trajectory starting in C6 and ending in C3. As expected, “transient” and “memory” OCRs exhibited robust IL-4-induced accessibility, and the former lost their induced accessibility, while the latter maintained accessibility after IL-4 washout; further, “primed” OCRs gained accessibility after IL-4 washout (Figure 5E and S5A). Next, we annotated the OCRs from each category to their putative target genes based on co-accessibility and proximity (Table S7). As expected, annotated genes also featured similar chromatin remodeling dynamics based on gene score values (e.g., *Arg1* – “transient”, *F7* – “memory”, *Atp6v0d2* – “primed”; Figure 5F). Finally, we analyzed “memory” OCRs by comparing their level of accessibility in the six MF clusters. This analysis demonstrated that both the establishment and stability of their accessibility was limited by CC (Figure 5G). These results identify “memory” OCRs in an IL-4-primed MF subset and show that CC prevents an increase in accessibility at these sites, extending CC regulation of MF behavior beyond the primary response to memory.

### IL-4 priming and cell cycle limits repolarization by IFNG at the chromatin level.

**Figure 6.**
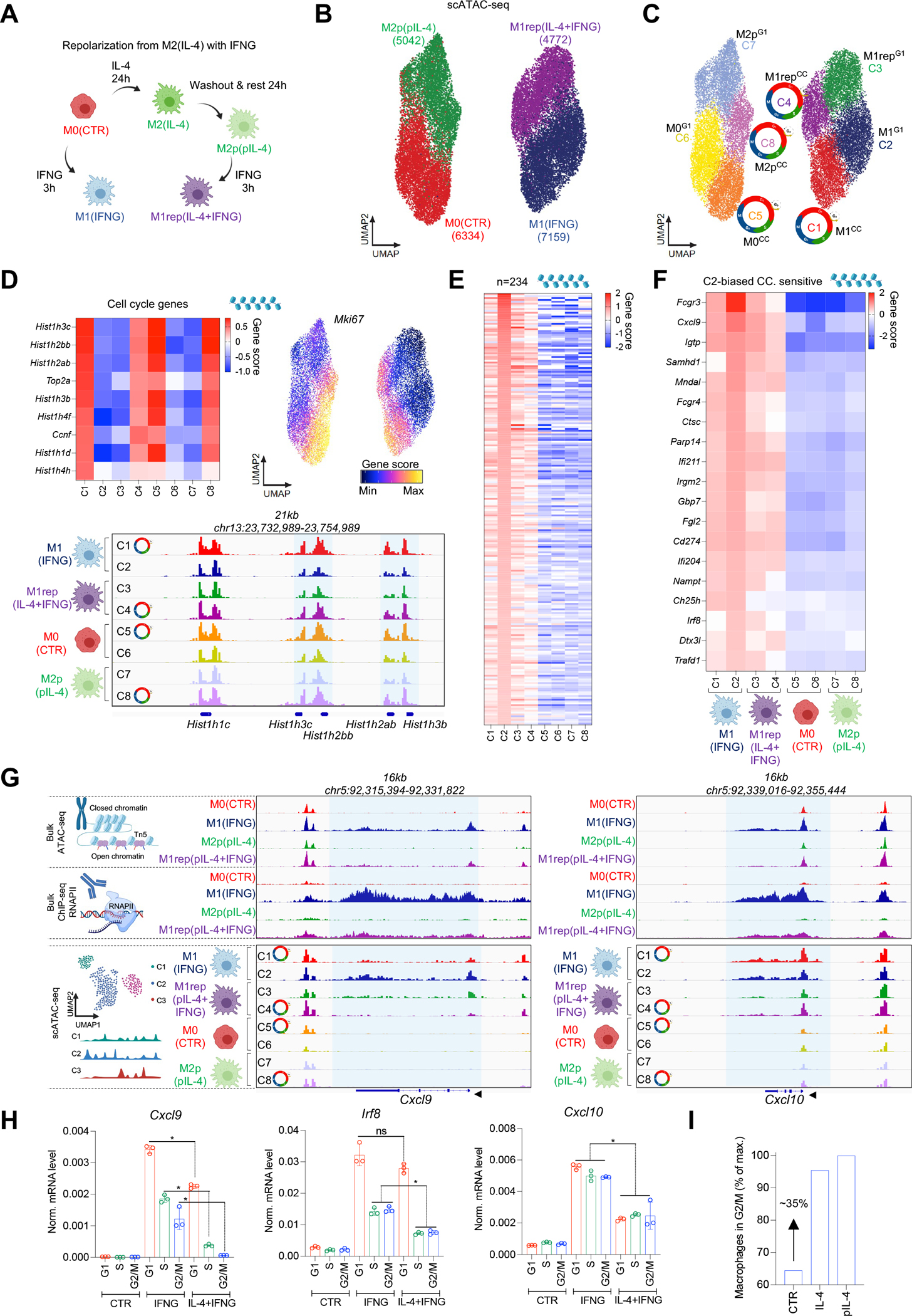
IL-4 priming and cell cycle limit the repolarization capacity of IFNG in a subset of MFs. **(A)** Scheme of repolarization. **(B)** scATAC-seq UMAP of MF conditions. **(C)** UMAP colored by the 8 chromatin state clusters. Cell cycle icons highlight cycling MF clusters. **(D)** Heatmap of cell cycle marker gene scores. UMAP of *Mki67* gene score values. Genome browser snapshot depicts scATAC-seq signal in cell cycle gene loci. **(E)** Gene score heatmap of cluster 2 markers across all clusters. **(F)** Gene score heatmap of the markers of cluster 2 that also show IFNG-induced, G1-phase-biased expression in bulk RNA-seq in Figure S4D. **(G)** Genome browser views depict bulkATAC-, RNAPII ChIP-, and scATAC-seq (8 clusters) signal in the *Cxcl9* and *Cxcl10* loci. **(H)** mRNA levels of *Irf8*, *Cxcl9* and *Cxcl10* in cell cycle from the indicated conditions. Statistics: two tailed, unpaired t-test at p<0.05, n=3. Data are represented as means ± SD. **(I)** Percentage of MFs in the G2/M phase of the cell cycle as determined by flow cytometry. Average of 3 experiments are used to calculate the percentage-wise distribution of cells in G2/M relative to the highest value (pIL-4).

To further test MF plasticity in CC, we used the experimental setting of repolarization.^20,23^ We performed IL-4-priming as previously described, but after the resting period, we exposed MFs to IFNG (repolarization) and performed scATAC- and scRNA-seq (Figure 6A). After projecting single-cell chromatin states from the 4 conditions in UMAP space (M0, M2p, M1, and repolarized – M1rep(pIL-4+IFNG)), we observed that M1rep MFs clustered next to M1 MFs and only slightly overlapped (Figure 6B). We identified 2 clusters in each condition, and again, clustering was driven by CC (8 clusters total; CC clusters - C1 – M1^CC^; C4 – M1rep^CC^; C5 – M0^CC^ and C8 – M2p^CC^; Figure 6C, D, S6A and S6B).

To determine the effects of CC and IL-4 priming in IFNG responsiveness, we defined all IFNG-induced chromatin remodeling events in M1^G1^ MFs (C2), and identified 234 marker gene scores, compared to all other MF clusters, suggesting that these genes exhibit reduced accessibility in cycling and/or IL-4 primed MFs (FDR≤0.01, Log_2_ FC≥1; Figure 6E, Table S8). Accordingly, M1rep^CC^ MFs (IL-4-primed MFs in CC - C4) displayed the most severe defect in chromatin opening at these sites upon IFNG stimulation, suggesting that CC and IL-4-priming together can induce an “IFNG-tolerant” MF plasticity state (Figure 6E). To better define the gene set that exhibits severely reduced IFNG responsiveness, we overlapped the IFNG-induced, CC-sensitive genes (Figure S4D; 41 genes – RNA-seq) with the marker gene scores (scATAC-seq accessibility) of M1^G1^ (C2) MFs, yielding genes with limited IFNG response in CC, and IL-4 priming as well (n=19; e.g., *Cxcl9*, and *Irf8*; Figure 6F). These results indicate that IL-4-priming and CC reduce MF plasticity to IFNG at the subpopulation level.

We tested this hypothesis with two CC-sensitive genes, *Cxcl9* and *Irf8*, and one CC-insensitive gene, *Cxcl10*. We visualized cluster-specific scATAC-seq signals together with bulk RNAPII ChIP-seq signal in repolarized MFs at the *Cxcl9* and *Cxcl10* gene loci (Figure 6G). As expected, IFNG-induced chromatin accessibility and RNAPII signal was reduced in M1rep MFs, compared to M1 MFs. Importantly, the two gene loci appeared to behave similarly in the bulk datasets; however, pseudo bulk scATAC-seq signal of each cluster revealed striking differences. *Cxcl9* exhibited reduced accessibility in CC (M1^CC^ - C1) and as a result of IL-4 priming (M1rep^G1^ - C3), but the combined effects of CC and IL-4 priming (M1rep^CC^ – C4) led to the lowest accessibility of *Cxcl9*, which was also supported by integrated scRNA-seq expression values (Figure 6G and S6A, B). We observed a similar pattern of chromatin accessibility change in the *Irf8* locus (Figure S6A, B). In contrast, *Cxcl10* was insensitive to CC, and we detected uniformly reduced chromatin accessibility/gene expression in IL-4-primed cycling (M1rep^CC^ - C4), compared to non-cycling MFs (M1rep^G1^ - C3; Figure 6G and S6B). We validated these findings by sorting M0, M1, and M1rep MFs from CC-phases and performing RT-qPCR (Figure 6H).

Prior studies have shown that IL-4 can induce MF proliferation *in vivo*;^38^ thus, we asked whether M2 and M2p MFs exhibit different CC-phase distributions, which may provide an additional mechanism for reduced IFNG responsiveness. We quantified the CC-phase distribution of M2 and M2p MFs by FACS and did not detect significant differences in the G1- or S-phases but observed a ~35% increase of MFs in the G2/M-phase in both conditions (Figure 6I and S6C). Thus, IL-4 priming can limit MF plasticity to IFNG not only via IL-4-induced epigenetic mechanisms, but also by changing the CC distribution of the population.

### MFs express a cell cycle-dependent tissue remodeling gene program during mouse tissue regeneration and human pulmonary fibrosis.

**Figure 7.**
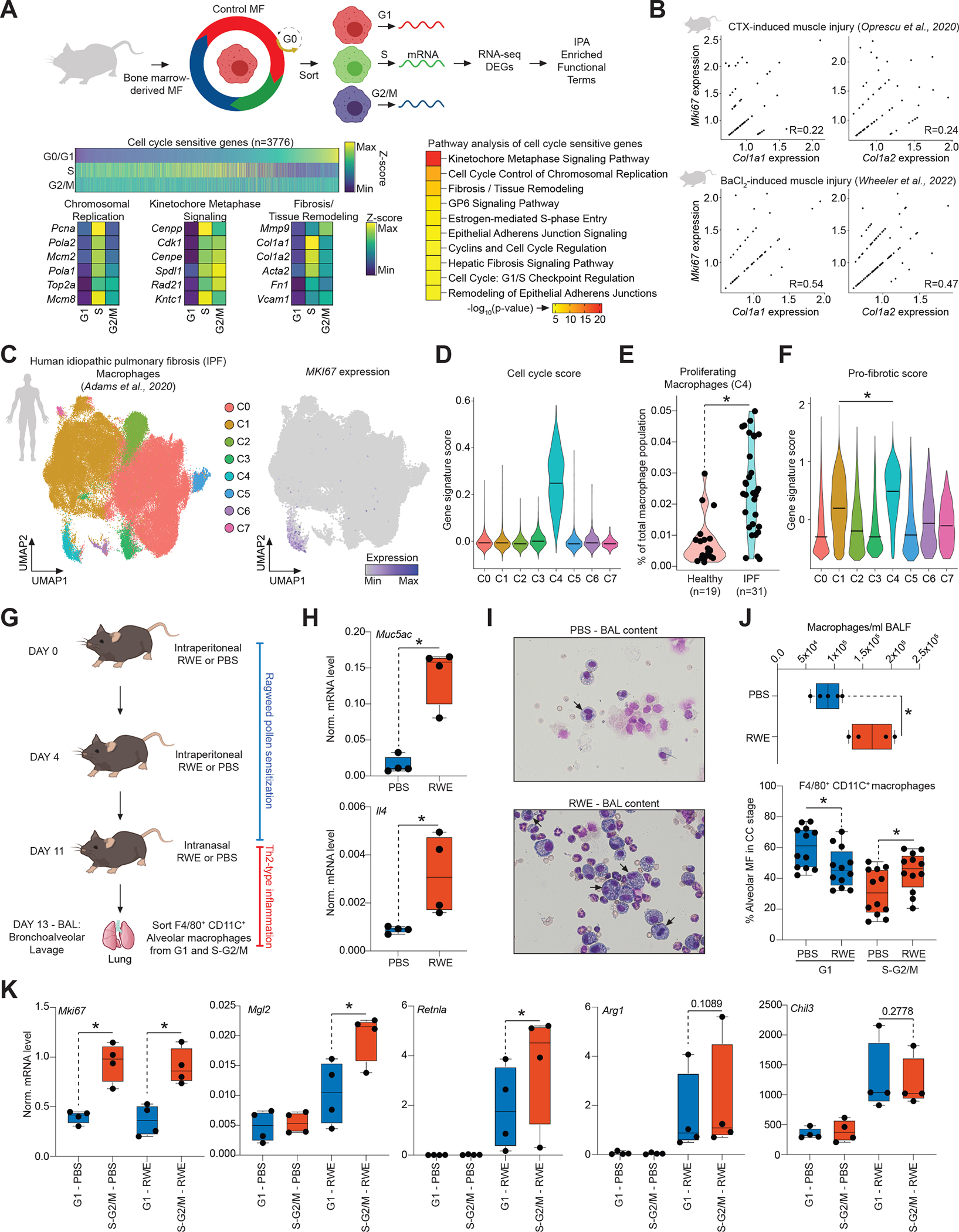
Cycling MFs express tissue regeneration genes. **(A)** Experimental scheme. DEGs – differentially expressed genes, IPA – Ingenuity Pathway Analysis. Heatmap represents DEGs across cell cycle phases in M0(CTR) MFs. IPA of the DEGs. Top 10 enriched biological functions are shown. Expression of a select set of genes from the first three enriched biological functions are shown as determined by bulk RNA-seq. **(B)** Feature scatter plots of the indicated gene pairs visualizing co-expression in single MFs (Log_2_Normalized Expression) with Pearson correlation coefficients. **(C)** scRNA-seq UMAP of human MFs from idiopathic pulmonary fibrosis (IPF) colored by the identified clusters. UMAP colored by the expression level of *MKI67*. **(D)** Violin plot represents the enrichment of a cell cycle gene signature score across clusters. **(E)** Violin plot represents the fraction of proliferating MFs in cluster 4 from control (Healthy) and IPF lungs. **(F)** Violin plot represents the enrichment of a pro-fibrotic gene signature score across MF clusters. Statistics: Wilcoxon Signed Rank Test at p<0.0001. **(G)** Scheme of the *in vivo* experimental system to induce Th2-type inflammation. **(H)** Boxplots depict the mRNA expression levels of the indicated genes. Statistics: two tailed, unpaired t-test at p<0.05, n=4. **(I)** Representative images of bronchoalveolar lavage (BAL) contents. **(J)** Quantification of MF numbers in the BAL fluid (n=4; top). Fraction of alveolar MFs in G1 and S-G2/M cell cycle phases in the indicated conditions. Statistics: two tailed, unpaired t-test at p<0.05, n=12. **(K)** Boxplots depict gene expression in alveolar MFs from cell cycle-phases in PBS or RWE-treated animals. Statistics: one tailed, paired t-test at p<0.05, n=4. All boxplots: box center line, median; limits, upper and lower quartiles; whiskers, 1.5× interquartile range.

Our findings show that CC alters MF plasticity to polarization signals but are there CC-intrinsic gene expression programs that might support specialized MF functions? To answer this question, we performed differential gene expression analysis across CC-phases in resting M0 MFs using bulk RNA-seq and identified 3,776 CC-sensitive and 7,327 insensitive genes (Benjamini–Hochberg adjusted p≤0.001; FC≥1.3) (Figure 7A and S7A). As expected, CC-sensitive genes were enriched for CC-related functional categories, but also for fibrosis- and tissue remodeling-related functional terms (e.g., Hepatic fibrosis/Tissue remodeling; Figure 7A), which exhibited S-G2/M-phase-biased expression (*e.g., Col1a1*, *Acta2*, and *Fn1*) (Figure S7B).

To investigate whether proliferating MF can express similar gene signatures *in vivo*, we analyzed two scRNA-seq datasets of murine muscle regeneration where MFs are indispensable for proper skeletal muscle regeneration: 1) a cardiotoxin (CTX)-induced model, and 2) a barium chloride (BaCl_2_)-induced *tibialis anterior* injury model.^43–47^ In both models, we observed proliferating MFs that expressed the tissue remodeling gene signature; moreover, at the single-cell level, a subset of proliferating MFs exhibited a weak positive correlation between the expression of CC genes and tissue remodeling genes (Figure 7B and S7C and S7D). Additionally, scoring MF subsets based on gene expression signatures of monocyte-derived and muscle-resident MF subsets in muscle regeneration suggested that both can be present and proliferate, and the latter are more likely to exist in a M2-like polarization state, than an M1 state, supporting our *in vitro* MF polarization data in CC (Figure S7C).^48^

Finally, to assess the potential human relevance of our findings, we reanalyzed a scRNA-seq dataset from human idiopathic pulmonary fibrosis (IPF; n=31) patients and control (n=19) lung donors.^49^ In IPF, MFs express strong pro-fibrotic gene programs and contribute to the progression of the disease.^49^ First, we identified MFs in the dataset (expressing e.g., *CD74* and *MRC1*), which comprised 7 distinct clusters, one of which was a proliferating MF cluster (cluster 4; Figure 7C and D). Second, we analyzed the numbers of proliferating MFs in cluster 4 from healthy and diseased samples, revealing more proliferating MFs (*MKI67*^+^) in diseased samples (Figure 7E). Finally, we found that proliferating MFs exhibited the strongest pro-fibrotic gene expression signature (e.g., *MMP9*, *FN1*, and *COL1A1*) among all MF subsets (Figure 7F). These results identify proliferating MFs that exhibit tissue remodeling-related phenotypes in mouse muscle regeneration and human IPF disease samples.

### Cyclical immune plasticity in tissue-resident alveolar macrophages during airway inflammation.

Finally, to assess whether cyclical immune plasticity can be detected in tissue-resident AMs, we used a Th2-type inflammation model, utilizing ragweed (RWE) pollen extract to induce airway inflammation in mice, mimicking an allergic reaction in the alveolar space. In this model, we sensitized the animals with intraperitoneal RWE injections, and induced airway inflammation by intranasal RWE exposure (Figure 7G). We observed severe Th2-type airway inflammation (induced *Muc5ac* and *Il4* expression) that led to increased AM presence (F4/80^+^ and CD11c^+^) in bronchoalveolar lavage samples (BAL), compared to control (PBS-treated). We sorted AMs from CC and measured the expression of *bona fide* Th2-type inflammatory genes (*Mgl2*, *Retnla*, *Arg1*, and *Chil3*). First, we determined the fraction of AMs in G1 vs S-G2/M, which showed an increase in AM proliferation after RWE treatment (Figure 7H–J and Figure S7E). Second, we measured the mRNA levels of Th2 inflammation-related genes. In agreement with induced *Il4* expression, all four genes showed robust upregulation in AMs of RWE-exposed animals, compared to the control group. Strikingly, we found that AMs expressed significantly more *Mgl2* and *Retnla* in the S-G2/M, while *Arg1* and *Chil3* expression were not significantly different between G1 and S-G2/M. As expected, *Mki67* showed strong S-G2/M-biased expression (Figure 7K). Therefore, cyclical plasticity operates in tissue-resident AMs that proliferate during Th2-type airway inflammation.

## Discussion

MF proliferation is a general phenomenon across tissues during an immune challenge, since entering CC replenishes and maintains MF populations;^17,38,50,51^ however, whether progression through the phases of CC also influences subsequent immunological functions/responses has not been investigated. Single-cell studies have generally relied on computational tools to remove CC signatures, prior to downstream data analysis, and thus, CC-associated phenotypic traits have previously been underexplored.^52^ Here, we exploit this feature of the dataset and provide experimental evidence that MFs engage biased polarization programs in CC. We provide evidence for CC-impacted MF responses in three different systems: (1) *in vitro* BMDM responses to polarization, priming, and repolarization, (2) *in vivo* AM responses to Th2-type inflammation, and (3) tissue regeneration and fibrosis during muscle injury or IPF. For example, *in vitro*, we report that the M1 polarization program is strongly restricted to the G1-phase of CC. M1 polarization has been reported to arrest MFs at the border of G1-S transition, perhaps to support the completion of the polarization process in agreement with our findings.^37^ In contrast, although ~50% of the IL-4-induced gene expression program is also G1-biased, a significant part of M2 polarization occurs in a S-G2/M-biased fashion, including *bona fide* polarization marker genes, such as *Chil3*, *Mgl2* and *Fn1*. Using CRISPR-editing in primary macrophages, we provide evidence that these gene expression patterns are regulated by phase-biased enhancer activities. These results suggest that IL-4-induced transcription and CC entry may synergize to achieve heterogeneous polarization states that are collectively known as the “M2 polarized” phenotype at the population level.^12,13,38^

Population-level analysis of the chromatin states underlying MF polarization revealed mostly transient chromatin remodeling events after the removal of polarization signals, although stability has been also noted.^22,23,28,53^ Our single-cell chromatin accessibility map recapitulated the mostly transient nature of the polarization process but identified a subset of “memory” MFs that retained an IL-4-induced chromatin imprint after cytokine removal, which was limited by CC. Several studies have employed opposing polarization signals and repolarization in this system to mimic MF responses in complex immunological microenvironments;^23,28,29^ however, these studies used bulk epigenome-mapping technologies and explained differences in MF responses solely by epigenetic effects that were established by the first stimuli. Our repolarization model uncovers dampened inflammatory response in IL-4 primed MFs and provide evidence that this can be achieved not only by an IL-4-driven epigenetic program, but also by herding MFs to the G2/M-phase that is not permissive to IFNG-induced transcription and can limit repolarization at the population level.

MFs proliferate in regenerating tissues after injury or infections, and recent studies noted the uncoupling of MF inflammatory and proliferative responses during infections of the lung and liver, connecting MF proliferation to resolution of inflammation and regeneration; however, no functional relevance has been linked to CC entry in terms of immunological function.^14–17^ Our work indicates that CC entry might provide a cyclical mechanism to dampen inflammation and support regeneration/maladaptive fibrotic responses as MFs can gain a tissue remodeling gene program in S-G2/M. Using published scRNA-seq datasets of regenerating mouse skeletal muscle and human IPF-diseased lung samples, we identified proliferating MF subsets, that express tissue remodeling/pro-fibrotic genes (e.g., *Col1a1, Mmp9, and Fn1*), further supporting this notion.^46,47,49,54^ These findings are compatible with the growing recognition that MF proliferation aligns with the reparative phase of tissue injury, and resolution of inflammation, putting forward the idea that MFs might obtain tissue regeneration-linked gene expression programs in CC, not merely the ones that support the execution of cell division.^14,16,17^ Finally, our *in vivo* allergic airway inflammation model also supports CC-gated Th2-inflammatory responses; though, in this system, AMs exhibit higher expression of two *bona fide* M2 marker genes, *Mgl2* and *Retnla* in CC.

In summary, our results demonstrate that CC is not only a driver of MF proliferation and self-renewal, but also of subsequent immunological responses to external stimuli. The gating of the MF response by CC may provide two advantages to the host organism. First, it may support the development of cyclical immune plasticity, whereby CC drives alternating inflammatory and regenerative gene expression programs that limit auto-inflammation and promote tissue repair. Second, dampened responses during CC may provide a mechanism to establish MF heterogeneity, for example, limiting excessive polarization and memory of the entire population to a single insult. Future studies should investigate whether additional proliferative immune cell types also use CC to tune their response to increase cellular heterogeneity and plasticity at the population level. Such responses across cell types may also impact therapeutic strategies targeting CC (e.g., CDK4/6 inhibitors), for example in cancer, and understanding the interplay between CC and immune activation may represent a promising avenue for therapeutic investigation.

## Limitations of the study

Although our results provide a few specific cases of CC phase-biased gene/enhancer activities, a more in-depth mechanistic understanding of CC-phase-biased gene expression is required. Additionally, we lack knowledge on the mechanism by which IL-4 priming, and CC obtain similar, negative effects on MF IFNG response. Further, we would like to call the readers’ attention that bone marrow-derived MF represent better a monocyte to MF differentiation system and might not necessarily mimic how tissue-resident MFs respond to environmental factors. Finally, the potential role of cycling MFs in tissue remodeling requires extensive future studies.

## STAR METHODS

### RESOURCE AVAILABILITY

#### Lead Contact

Further information and requests for resources and reagents should be directed to the lead contact Ansuman T. Satpathy (satpathy@stanford.edu).

#### Materials Availability

Materials that are used in the study are commercially available and are reported in the STAR METHODS Key Resources Table.

#### Data and Code Availability

- Sequencing data has been deposited to GEO under accession: GSE178526. Published data that has been used in this study: GSE138826, GSE84520, and GSE136831.
- Code that was used to analyze the presented single-cell datasets is available at: DOI: 10.5281/zenodo.7332328.
- Any additional information required to reanalyze the data reported in this paper is available from the lead contact upon request.

### EXPERIMENTAL MODEL AND SUBJECT DETAILS

#### Bone marrow-derived macrophage culture

Wild type, 2–3 months old female C57/Bl6 mice were purchased from Jackson laboratories. All mice were housed in a specific-pathogen-free facility and were used for experiments at 8–12 weeks of age. All experiments were performed according to protocols approved by the Institutional Animal Care and Use Committees of Stanford University (protocol number: 33814). Mice were sacrificed and bone marrow was isolated form the tibiae and femora of the animals. Red blood cell lysis was carried out and cells were plated in differentiation media containing 10% FBS, Dulbecco’s Modified Eagle’s Medium (DMEM) and 20ng/ml mouse M-CSF (Peprotech). On the third day of differentiation, media was replaced with fresh differentiation media. Cytokine treatments, sorting procedures were carried out on the 6^th^ day of differentiation.

#### Treatment conditions

Macrophages were treated with either IL-4 (20ng/ml) or IFNG (20ng/ml) (Peprotech). For the single-cell polarization experiments, we used 24-hours of IL-4 polarization and 3 hours of IFNG polarization in macrophage differentiation media that contained 10% FBS, 1% penicillin/streptomycin and M-CSF (20ng/ml). IL-4 priming was performed as follows: macrophages were polarized with IL-4 (20ng/ml) for 24-hours. Cells were washed three times with serum-free DMEM, and then, differentiation media was replaced, and cells were rested for an additional 24-hours. Repolarization was performed at this point for 3-hours with IFNG (20ng/ml). Macrophage polarization for cell cycle experiments used 3-hours of polarization with either IL-4 or IFNG (both at a 20ng/ml concentration). For the cell cycle inhibitor experiments, macrophages were pretreated for 24-hours with DMSO/EtOH (Vehicle), Ribociclib (Ribo. – 1μM) or Artesunate (Arte. – 10μM) followed by 3-hours of polarization with either IL-4 (20ng/ml) or IFNG (20ng/ml). Experiments of CRISPR-Cas9 enhancer engineering were conducted with 3-hour long IL-4 treatments, 5 days after differentiation (IL-4 final concentration 20ng/ml).

### METHODS DETAILS

#### CRISPR-Cas9 enhancer editing

Bone marrow-derived cells were isolated from the tibiae and femora of female animals, and then, were subjected to electroporation (LONZA - 4D Nucleofactor) with Cas9, complexed with guide RNAs that target the STAT6 motif within the enhancers of *Retnla* and *Mgl2*, or target a non-relevant gene promoter (*Mrc1*). Briefly, crRNA (80μM) and tracRNA (160μM) were ordered from Integrated DNA Technologies (IDT) and were annealed in a 2:1 ratio in duplex buffer at 37C for 30-minutes to generate sgRNAs. sgRNAs were then complexed with recombinant Cas9 protein in a 2:1 ratio at 37C for 15-minutes to generate Cas9-RNP (ribonucleoprotein). Approximately, 5×10^5^ cells were resuspended in P3-buffer and electroporated with Cas9-RNP with the following program: ED113. Cells were then plated onto 6-well plates in macrophage differentiation media in the presence of 20ng/ml M-CSF and were differentiated for 5-days (three technical replicates were used that were pooled from 5 mice). On day 5, cells were polarized with IL-4 (20ng/ml) for 3-hours, and then, cells were stained with Vybrant Dye Cycle (as described in the FACS methods section) to sort from cell cycle-phases. Cell cycle sorted macrophages were lysed in Trizol for RNA isolation. Guide RNA sequences are available from Table S10.

#### Phagocytosis assays

Macrophages were differentiated in 6 well plates, each well having approximately 10^6^ cells. On the 6^th^ day of differentiation, macrophages were treated with bacterial conjugates (pHrodo green E. Coli and pHrodo red S. Aureus - Invitrogen) by adding 40μl particles into 4ml macrophage differentiation media. Macrophages were incubated with the particles for 2 hours at 37C; then, cells were collected by scraping and Vybrant Dye Cycle was added in a 1:500 dilution, followed by 30 minutes incubation at 37C in Eppendorf tubes. Propidium iodide was added in the last five minutes, and then, cells were subjected to FACS analysis.

#### Animal sensitization and challenge

Six- to 8-week-old female C57/B6 WT mice were used for these studies. Allergic airway inflammation was induced with endotoxin-free ragweed pollen extract (RWE, Greer Laboratories; catalog number: XP56D3A2.5) as we previously described with some modification.^55^ Briefly, animals were sensitized with two intraperitoneal (i.p.) administrations (on day 0 and 4) of 300 μg RWE in calcium and magnesium free Dulbecco’s phosphate-buffered saline (PBS, Sigma; catalog number: D8537) injection combined in a 3:1 (75 μl:25 μl) ratio with alum adjuvant (Thermo Fischer Scientific, Waltham, MA, USA; catalog number: 77161) or injected with the same volumes of PBS, as a vehicle control. On day 11, parallel groups of mice were challenged intranasally under ketamine and xylazine sedation with 240μg RWE dissolved in 60 μl of phosphate-buffered saline or same volumes of PBS. On day 13, animals were handled according to the regulatory standards of the animal facilities of the University of Debrecen. Animal studies were approved by the Animal Care and Protection Committee at the University of Debrecen (16/2019/DE MAB).

#### Bronchoalveolar lavage sample preparation

Bronchoalveolar lavage (BAL) fluid was performed on day 13, after the last intranasal challenge. To collect BALF samples, animals were euthanized, and their tracheas were cannulated. Lavage was performed with 2 aliquots of 0.7 ml of ice-cold PBS (pH 7.3). The BALF cells were centrifuged at 800 g for 10 minutes at 4 °C and the supernatants were removed. Collected BALF cells were used for cell sorting and cell counting.

#### Fluorescence-activated cell sorting (FACS)

Macrophages (~3 × 10^6^) were treated with Fc-block (1:100) for 15-minutes on ice, and then, stained with anti-F4/80 (rat monoclonal FITC-conjugated, BioLegend) in a 1:200 dilution in FACS buffer for 20 minutes on ice. Cells were spun and resuspended in serum-free DMEM pre-heated to 37C with Vybrant DyeCycle Violet Stain (1:500) and incubated for 30 minutes at 37C followed by Propidium Iodide (PI) staining and sorting. PI negative F4/80 positive macrophages were sorted from all three cell cycle stages according to the Vybant DyeCycle signal. Cell surface staining of macrophages were done as follows: approximately, 10^6^ macrophages were differentiated in 6 well plates, cells were treated for 24 hours with IL-4 (final concentration - 20ng/ml), and then cells were treated with Fc-block (1:100), and stained for MGL2 – PE-Cy7 and MRC1 – APC in a 1:200 dilution for 30 minutes on ice in FACS buffer, followed by Aqua live/dead staining for 15 minutes on ice. For intracellular staining (ICS) with RETNLA, RETNLA antibody (PeproTech – 500-P214) was first conjugated to APC fluorophore with the Zenon APC Rabbit IgG labeling kit according to the manufacturer’s protocol (Thermo Fisher Scientific – Z25303). For ICS, Foxp3 transcription factor staining buffer set was used (eBioscience – 00-5523-00), and the protocol provided by the manufacturer was followed. Briefly, cells were stained with Aqua live/dead stain (Thermo Fisher Scientific – L34957), then, cells were fixed in IC Fixation Buffer, and were incubated for 1-hour at room temperature. Cells were washed in 1X Permeabilization Buffer, and after centrifugation, cell pellets were resuspended in 100ul 1X Permeabilization Buffer, containing 5ul of freshly conjugated RETNLA - APC antibody, and then, were incubated at room temperature for 1-hour followed by FACS analysis.

BALF samples were collected, and single cell suspensions were prepared in FACS buffer containing 0.5% BSA and 2 mM EDTA in sterile PBS. Cells were incubated with FcR Blocking Reagent (Miltenyi Biotec), F4/80-APC (clone BM8, BioLegend) and CD11c-PE (clone HL3, BD Pharmingen) antibody conjugates for 20 minutes on ice in dark followed by washing step with FACs buffer. To discriminate live and dead cells, the LIVE/DEAD^™^ Fixable Green Dead Cell Stain Kit (Thermo Fischer Scientific) was used based on the manufacturer’s recommendation. To measure cell cycle phases, cells were incubated with Vybrant^™^ DyeCycle^™^ Violet Stain (Thermo Fischer Scientific) in high glucose DMEM culture media for 30 minutes at 37°C in humidified environment containing 5% CO_2_. The flow cytometry analysis and cell sorting were performed by BD FACSAria III (BD Biosciences) using BD FACSDiva Software 6.0 (BD Biosciences). The acquired flow cytometry data were analyzed with FlowJo v10 (BD Biosciences).

#### Cell counting in BALF samples

Total cell counts in BAL were determined from an aliquot of the cell suspension. Differential cell counts were performed on cytocentrifuge preparations stained with eosin and thiazine (ELITech Biomedical Systems: Red Stain Reagent, catalog number: SS-035C-EU; Blue Stain, catalog number: SS-035/049B-EU, Rinse, catalog number: SS-035A-EU, Aerofix Additive, catalog number: SS-148-EU).

#### Assessment of Muc5ac and Il-4 expression in the lungs

Expression levels of Muc5ac and Il-4 were analyzed by Real-Time Quantitative PCR mRNA detection (qPCR). RNA was isolated from a half of frozen mouse lung with Trizol reagent (Zymo Research). RNA was reverse transcribed with High-Capacity cDNA Reverse Transcription Kit (Applied Biosystems) according to manufacturer’s protocol. Transcript quantification was performed by qPCR reactions using SYBR green master mix (Bio-Rad Laboratories). Transcript levels were normalized to *Ppia*.

#### Real-time quantitative PCR for enhancer RNA and mRNA detection (qPCR)

RNA was isolated with Trizol reagent (Ambion). RNA was reverse transcribed with High-Capacity cDNA Reverse Transcription Kit (Applied Biosystems) according to the manufacturer’s instructions. Transcript quantification was performed by qPCR reactions using SYBR green master mix (BioRad). Transcript levels were normalized to *Ppia*. Primer sequences are available from Table S10.

#### Chromatin immunoprecipitation sequencing (ChIP-seq)

ChIP-seq was performed as previously described with minor modifications.^56^ Bone marrow-derived macrophages (3 × 10^6^) were double crosslinked by 50mM DSG (disuccinimidyl glutarate, #C1104 - ProteoChem) for 30 minutes followed by 10 minutes of 1% formaldehyde. Formaldehyde was quenched by the addition of glycine. Nuclei were isolated with ChIP lysis buffer (1% Triton x-100, 0.1% SDS, 150 mM NaCl, 1mM EDTA, and 20 mM Tris, pH 8.0). Nuclei were sheared with Covaris sonicator using the following setup: Fill level – 10, Duty Cycle – 5, PIP – 140, Cycles/Burst – 200, Time – 4 minutes). Sheared chromatin was immunoprecipitated with RNAPIIpS2 antibody (Abcam - ab5095). Antibody chromatin complexes were pulled down with Protein A magnetic beads and washed once in IP wash buffer I. (1% Triton, 0.1% SDS, 150 mM NaCl, 1 mM EDTA, 20 mM Tris, pH 8.0, and 0.1% NaDOC), twice in IP wash buffer II. (1% Triton, 0.1% SDS, 500 mM NaCl, 1 mM EDTA, 20 mM Tris, pH 8.0, and 0.1% NaDOC), once in IP wash buffer III. (0.25 M LiCl, 0.5% NP-40, 1mM EDTA, 20 mM Tris, pH 8.0, 0.5% NaDOC) and once in TE buffer (10 mM EDTA and 200 mM Tris, pH 8.0). DNA was eluted from the beads by vigorous shaking for 20 minutes in elution buffer (100mM NaHCO_3_, 1% SDS). DNA was de-crosslinked overnight at 65C and purified with MinElute PCR purification kit (Qiagen). DNA was quantified by Qubit and 10 ng DNA was used for sequencing library construction with the Ovation Ultralow Library System V2 (Tecan) using 12 PCR cycles. Libraries were sequenced on an Illumina Hiseq 2500 using paired-end 75bp reads.

#### Bulk ATAC-seq and ChIP-seq computational methods

Bulk epigenetics datasets were analyzed as described previously.^57^ Briefly, reads were trimmed for quality and adapter sequences using fastp. Trimmed reads were aligned to the mm10 reference genome using hisat2. Aligned reads were deduplicated using picard. Peaks were called for each sample using MACS2. A fixed-width, reproducible union peak set for each group of samples (e.g., bulk ATAC-seq samples) was constructed by iteratively merging individual peak calls for each sample and removing overlapping peaks until a final, non-overlapping set of peaks was obtained. The union peak set was used to create a sample by peak matrix. ATAC-seq coverage tracks were obtained by exporting normalized bigwig files from R, normalized to reads in TSS, a gold-standard normalization method that controls for both sequencing depth and library quality.^58^

#### Bulk RNA-seq

Approximately 20ng total RNA was used for library preparation with Ovation Ultralow RNA-seq V2 (Tecan) from two biological replicates. Libraries were generated according to the manufacturer’s instructions. Approximately 50ng amplified cDNA was subjected to Ovation Ultralow V2 library generation and manufacturer’s instructions were followed. Libraries were size selected with E-Gel EX 2% agarose gels (Life Technologies) and purified by QIAquick Gel Extraction Kit (Qiagen). Libraries were sequenced on HiSeq 2500 instrument.

#### RNA-seq analysis

Fastq files were pseudoaligned to a mm10 transcriptome index and the abundance of transcripts was quantified using Kallisto v0.43.1 with bias correction.^59^ The transcript-level abundance estimates were imported and summarized using tximport v1.16.1, and differential expression was determined using the DESeq2 package v1.28.11 in Bioconductor v.3.11. A gene was considered cell cycle-sensitive if it was differentially expressed between any two cell cycle stages in the control condition or the condition of interest (IL-4, or IFNG respectively) with an absolute fold change of ≥1.3 and a Benjamini–Hochberg adjusted p-value ≤0.001. If a gene was not differentially expressed between any two cell cycle stages with an adjusted p-value ≤0.001, it was considered cell cycle-insensitive. The cell cycle stage bias of a gene was assigned to the cell cycle stage where the gene showed the largest absolute scaled variance-stabilizing transformed expression.

#### scATAC-seq sample and library generation

Single cell ATAC-seq experiments were performed on the 10x Chromium platform as described earlier.^31^ Briefly, after cytokine treatments, macrophages were subjected to nuclei isolation according to the protocol of the manufacturer. Nuclei were counted and ~20,000 were submitted for tagmentation. After tagmentation, nuclei were loaded for capture using the 10x Chromium controller. After Gel emulsion generation, linear amplification was performed, followed by DNA purification following the manufacturer’s protocol. The resulting DNA was used for library construction as described on the website of the manufacturer. Libraries were quantified by quantitative PCR and were sequenced on an Illumina Hiseq 2500 sequencer, using the following setup: 50bp read 1N, 8bp i7 index, 16bp i5 index and 50bp read 2N. In this reaction, 1N and 2N refers to the DNA insert sequencing, while i5 and i7 sequencing identifies the individual barcodes of single cells.

#### Single-cell RNA-seq library preparation

Single-cell RNA-seq libraries were prepared using the 10X Single Cell Immune Profiling Solution Kit (v1 Chemistry), according to the manufacturer’s instructions. Briefly, FACS sorted cells were washed once with PBS + 0.04% BSA. Following reverse transcription and cell barcoding in droplets, emulsions were broken, and cDNA purified using Dynabeads MyOne SILANE followed by PCR amplification (98°C for 45 sec; 14 cycles of 98°C for 20 sec, 67°C for 30 sec, 72°C for 1 min; 72°C for 1 min). For gene expression library construction, 50 ng of amplified cDNA was fragmented, and end-repaired, double-sided size selected with SPRIselect beads, PCR amplified with sample indexing primers (98°C for 45 sec; 14 cycles of 98°C for 20 sec, 54°C for 30 sec, 72°C for 20 sec; 72°C for 1 min), and double-sided size selected with SPRIselect beads. Single-cell RNA libraries were sequenced on an Illumina HiSeq 4000 to a minimum sequencing depth of 25,000 reads/cell using the read lengths 28bp Read1, 8bp i7 Index, 91bp Read2.

#### scATAC-seq computational methods

scATAC-seq datasets were processed as described previously.^60^ Briefly, reads were filtered, trimmed, and aligned to the mm10 reference genome using the 10X cellranger atac-count pipeline. Fragments files were loaded into ArchR for additional processing and analysis.^32^ Separate ArchR projects were created for the three sample sets (priming, polarization, and repolarization) and additionally for each individual sample. Doublets were identified and removed using ArchR’s default doublet simulation and calling procedures. Barcodes were removed that had an enrichment of Tn5 insertions in transcription start sites (TSS enrichment) less than 4 or less than 1000 fragments. Tiles and GeneScores matrices were computed by summing Tn5 insertions in predefined genomic windows. After clustering the cells, peaks were called by macs2 on pseudoreplicates sampled from each cluster to obtain a reproducible peak set retaining cell type specific peaks. Transcription factor motif deviations were computed using chromVar.^61^ Imputation was performed using Magic.^62^ Pseudo-bulk tracks for indicated groups of cells were exported from ArchR as bigwig files normalized by reads in transcription start sites. Tracks were visualized in the Integrative Genomics Viewer (IGV). ArchR computes peak co-accessibility from a peak matrix. ArchR was used to identify low-overlapping cell aggregates (more than 500 cells, as described in Granja et al., 2021),^32^ and then, for each chromosome, cell aggregate by peak matrix was read in by ArchR. All possible peak-to-peak combinations were identified within a 250kb window, and Pearson correlation of the log_2_-normalized cell aggregate by peak matrix was calculated. Column sums across all chromosomes were used for depth normalization. This procedure was done for all chromosomes, and the combined, genome-wide results were stored in the ArchRProject that can be accessed for downstream applications. Peak co-accessibility for the identified distant open chromatin regions in Figure 5D was computed by ArchR’s addCoAccessibility function, which stores peak co-accessibility information in the ArchRProject. The resulting peak pairs were further screened for the identified chromatin behaviors (“Transient”, “Memory”, and “Primed”). OCRs that exhibited these chromatin behaviors were annotated to genes based on the following criteria: +/− 250kb window around the transcription start sites (TSSs) of the genes that had co-accessible promoters (defined as peaks overlapping with a +/− 1kb genomic window around the TSSs of genes). Results of gene-OCR pairs are available from Table 7. Trajectory analyses was performed using ArchR’s addTrajectory and plotTrajectory functions, and clusters were manually defined as starting and end points of the trajectory to recapitulate potential macrophage polarization fates across conditions.

#### scRNA-seq computational methods

Reads were filtered, trimmed, and aligned to the mm10 reference genome using the 10X cellranger count pipeline. Additional analysis was performed in R using Seurat with default parameters.^65^ Doublets were called for each sample individually using the R implementation of scrublet,^63^ rscrublet. Gene by barcode counts matrices were loaded into Seurat for additional processing and analysis.^64^ Separate Seurat objects were created for the three sample sets (priming, polarization, and repolarization) and for each individual sample. Barcodes with >12.5% mitochondrial reads, <200 unique features, or a scrublet score >0.25 were removed. Remaining cells were then clustered and visualized.

Cell cycle phase predictions for each cell were performed following the vignette available online: https://satijalab.org/seurat/archive/v3.1/cell_cycle_vignette.html, according to Tirosh et al., 2016.^33^

Published datasets were also analyzed according to the following standards: Gene by barcode raw counts matric of single-cell datasets of IPF patients are retrieved from GSE136831. Doublets were removed by Scrublet and the processed matrix was loaded into Seurat. ‘Macrophage’ and ‘Alveolar Macrophage’ clusters were subset by ‘Manuscript_Identity’ metadata in the original dataset from downstream analysis.^49^ Batch effects between IPF and control groups are removed with Harmony. Cells were then clustered and visualized using FindClusters() with 1:30 Principal Components (PCs) and resolution = 0.1. Cell cycle scores were added with CellCycleScoring() with default marker genes. Pro-fibrotic score was calculated by *FN1*, *MMP9*, *COL1A1*, *COL22A1*, and *CCL18* expression with AddModuleScore() function according to Adams et al., 2020.^49^ Module scores were also calculated for M1 and M2 polarization signatures using the top 20 M1 and M2 polarization-specific induced genes from scRNA-seq. Additionally, skeletal muscle-resident and monocyte-derived macrophage module scores were calculated based on the gene signatures reported by Wang et al., 2020.^48^

### QUANTIFICATION AND STATISTICAL ANALYSIS

Statistical analyses were performed in R or GraphPad Prism. qPCR measurements were presented as means +/− SD and three biological replicates were performed. The exact replicate numbers are indicated in the figure legends for each experiment. On the bar graphs, significant changes were determined by two tailed, unpaired t-test at p<0.05. Differential chromatin accessibility analyses across cell clusters were performed with the following parameters: FDR ≤0.01, Log_2_ FC≥1.25, unless specified otherwise. Differential gene expression analyses of scRNA-seq results were performed with the following parameters: FDR≤0.01, FC≥1.3. Cell cycle phase-biased gene expression levels were determined as follows: Benjamini–Hochberg adjusted p-value ≤0.001; FC≥1.3 (two biological replicates were used). Significant changes between the median peak scores of “Transient”, “Memory” and “Primed” chromatin regions were determined by Wilcoxon Signed Rank Test, p<0.0001. Statistical parameters are reported in the figure legends and in the results section.

## Supplementary Material

Supplemental figures

Table S1 Table 1: Gene scores of the trajectory analysis of M2 MF polarization. Related to Figure 1.

Table S2 Table 2: Motif deviation scores of the trajectory analysis of M2 MF polarization. Related to Figure 1.

Table S3 Table 3: Gene scores of the trajectory analysis of M1 MF polarization. Related to Figure 1.

Table S4 Table 4: Motif deviation scores of the trajectory analysis of M1 MF polarization. Related to Figure 1.

Table S5 Table 5: Cluster-biased gene score values of polarized MFs. Related to Figure 2.

Table S6 Table 6: Marker genes of M1 and M2 MFs determined by scRNA-seq. Related to Figure S2B.

Table S7 Table 7: Peak scores with transient, memory and primed kinetics. Related to Figure 5.

Table S8 Table 8: Cluster-biased gene score values of repolarized macrophages. Related to Figure 6.

Table S9 Table 9: Z-scores of cell cycle sensitive genes in M1 and M2 MFs. Related to Figure 4.

Table S10 Table 10: Primer/sgRNA sequences used in this study. Related to Figure 4.

## Figures and Tables

**Key resources table:** 

REAGENT or RESOURCE	SOURCE	IDENTIFIER
**Antibodies**		
Anti-F4/80 – FITC	BioLegend	(BioLegend Cat# 123108, RRID:AB_893502)
Anti-MGL2(CD310b) – PE-Cy7	BioLegend	(BioLegend Cat# 146807, RRID:AB_2563389)
Anti-MRC1(CD206) – APC	BioLegend	(BioLegend Cat# 141708, RRID:AB_10900231)
Anti-RETNLA(RELMa) - Unconjugated	PeproTech	(PeproTech Cat# 500-P214-50ug, RRID:AB_1268843)
Anti-CD16/CD32	eBioscience	RRID:AB_467133
Anti-F4/80 – APC	BioLegend	(BioLegend Cat# 123108, RRID:AB_ 893493)
Anti-Cd11c – PE	BD Bioscience	(BD Biosciences Cat# 553802, RRID:AB_395061)
RNAPII-pS2	Abcam	Abcam Cat# ab5095, RRID:AB_304749
**Biological samples**		
Mouse bone marrow samples	Stanford University	N/A
Mouse bronchoalveolar lavage samples	University of Debrecen	N/A
**Chemicals, peptides, and recombinant proteins**		
Mouse IL-4	PeproTech	214-14
Mouse IFNG	PeproTech	315-05
Mouse M-CSF	PeproTech	315-02
Ribociclib	Selleck Chemicals	S7440
Artesunate	Tocris	6827
Ragweed pollen extract	Greer Laboratories	XP56D3A2.5
Alum Adjuvant	Thermo Fisher Scientific	77161
Cas9 Nuclease V3	IDT	N/A
Red Stain Reagent	ELITech Biomedical Systems	SS-035C-EU
Blue Stain Reagent	ELITech Biomedical Systems	SS-035/049B-EU
Rinse	ELITech Biomedical Systems	SS-035A-EU
Aerofix Additive	ELITech Biomedical Systems	SS-148-EU
Disuccinimidyl Glutarate (DSG)	ProteoChem	C1104
Formaldehyde	Thermo Scientific	28906
Trizol	Thermo Fisher Scientific	15596018
**Critical commercial assays**		
Ovation Ultralow System 2	Tecan	0344NB-A01
Ovation RNA-seq System V2	Tecan	7102-32
10x scRNA-seq (v1.1 chemistry)	10x Genomics	1000165
10X scATAC-seq (v1 chemistry)	10x Genomics	1000175
pHrodo Red S. Aureus	Thermo Fisher Scientific	A10010
pHrodo Green E. coli	Thermo Fisher Scientific	P35366
Vybrant DyeCycle Violet Stain	Thermo Scientific	V35003
P3 Primary Cell 4D X Kit S (32 RCT)	Lonza	V4XP-3024
Zenon APC Rabbit IgG labeling kit	Thermo Fisher Scientific	Z25303
FOXP3 transcription factor staining buffer set	eBioscience	00-5523-00
Live/dead Fixable Aqua Dead Cell Stain Kit	Thermo Fisher Scientific	L34957
LIVE/DEAD Fixable Green Dead Cell Stain Kit	Thermo Fisher Scientific	L23101
High-Capacity cDNA Reverse Transcription Kit	Thermo Fisher Scientific	4368814
SsoAdvanced Universal SYBR Green Supermix	BioRad	1725274
MinElute PCR Purification Kit	Qiagen	28006
**Deposited data**		
Raw and analyzed data.	This paper.	GEO: GSE178526
Original code	This paper.	DOI: 10.5281/zenodo.7332328
**Experimental models: Cell lines**		
Primary bone marrow-derived macrophages (BMDMs)	Female C57BL/J6	N/A
Primary alveolar macrophages	Female C57BL/J6	N/A
**Experimental models: Organisms/strains**		
Mouse: C57BL/J6	The Jackson Laboratory	IMSR_JAX:000664
**Oligonucleotides**		
Guide RNA sequences, see Table	This paper. IDT	Table 10
Primer sequences, see Table	This paper. IDT	Table 10
TracrRNA	IDT	1072533
**Software and algorithms**		
BD FACSDiva Software	BD Biosciences	RRID:SCR_001456
FlowJo	BD Biosciences	RRID:SCR_008520
GraphPad Prism	SciCrunch Registry	RRID:SCR_002798
Seurat	Hao et al,^65^	https://satijalab.org/seurat/index.html
Cellranger	10X Genomics	https://support.10xgenomics.com/singlecell-gene-expression/software/pipelines/latest/using/count
ArchR	Granja et al,^32^	ArchR (RRID:SCR_020982)
Bioconductor	SciCrunch Registry	Bioconductor (RRID:SCR_006442)
DESeq2	SciCrunch Registry	DESeq (RRID:SCR_000154)
Kallisto	SciCrunch Registry	kallisto (RRID:SCR_016582)
Tximport	SciCrunch Registry	tximport (RRID:SCR_016752)
ChromVAR	Schep et al,^61^	https://bioconductor.org/packages/release/bioc/html/chromVAR.html
Magic	Van Dijk et al,^62^	https://github.com/KrishnaswamyLab/MAGIC
Scrublet	SciCrunch Registry	Scrublet (RRID:SCR_018098)
Harmony	SciCrunch Registry	Harmony (RRID:SCR_022206)
Fastp	SciCrunch Registry	fastp (RRID:SCR_016962)
Hisat2	SciCrunch Registry	HISAT2 (RRID:SCR_015530)
MACS2	SciCrunch Registry	MACS (RRID:SCR_013291)
BEDTools	SciCrunch Registry	BEDTools (RRID:SCR_006646)
Bestus Bioinformaticus Tools	SciCrunch Registry	Bestus Bioinformaticus Tools (RRID:SCR_016968)

## Highlights

- Single-cell chromatin maps reveal heterogeneous MF polarization states
- Heterogeneous MF responses to polarizing cytokines coincides with cell cycle
- MF polarization is a cell cycle phase-dependent immunological process
- MF gene expression in S-G2/M is linked to tissue remodeling

## INCLUSION AND DIVERSITY

We support inclusive, diverse, and equitable conduct of research.
