# Supplementary material for: Macrophage inflammatory and regenerative response periodicity is programmed by cell cycle and chromatin state
Source: Mol Cell. Author manuscript; Available in PMC 2024 Jan 5. (PMC9831293; doi:10.1016/j.molcel.2022.11.017)
Supplement: Supplemental figures [file NIHMS1855552-supplement-Supplemental_figures.pdf]

MOLECULAR-CELL-D21-01410

Supplemental information

Macrophage inflammatory and regenerative response periodicity is programmed by cell cycle and chromatin state

Bence Daniel, Julia A. Belk, Stefanie L. Meier, Andy Y. Chen, Katalin Sandor, Zsolt Czimmerer, Zsofia Varga, Krisztian Bene, Frank A. Buquicchio, Yanyan Qi, Hugo Kitano, Joshua R. Wheeler, Deshka S. Foster, Michael Januszyk, Michael T. Longaker, Howard Y. Chang and Ansuman T. Satpathy

Supplementary Figure 1.

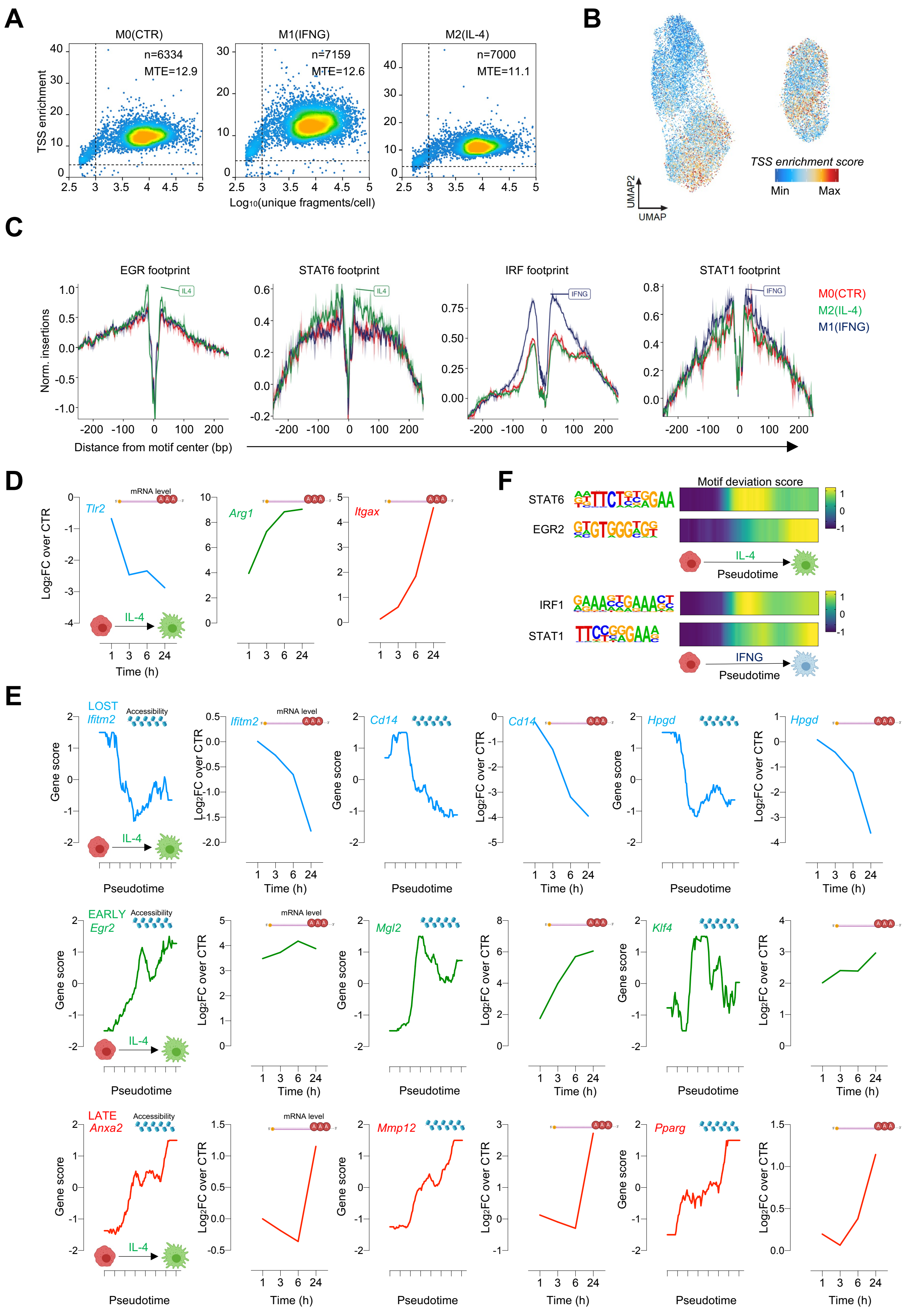

Figure S1. Single cell chromatin accessibility landscape of MF polarization. Related to Figure 1.

(A) Density plot of transcription start site (TSS) read enrichment as a function of unique scATAC fragment count per cell for the three samples. Cells passing the filter of having at least a TSS enrichment score of 3 and 1000 unique fragments are used in downstream analyses. Median TSS enrichment (MTE) is shown for each sample. **(B)** UMAP of TSS enrichment scores. **(C)** Tn5 bias corrected transcription factor footprints in M0(CTR), M2(IL-4) and M1(IFNG) macrophages around the respective motif's center. **(D)** Relative expression level of *Tlr2*, *Arg1* and *Itgax* from an IL-4 time course bulk RNA-seq experiment (GSE106706). **(F)** Heatmap visualization of the motif deviation scores of the indicated transcription factors over the M0(CTR) – M2(IL-4) and M0(CTR) – M1(IFNG) polarization trajectories. **(E)** scATAC-seq gene score values over the M2 polarization trajectory and relative bulk RNA-seq expression values (GSE106706) over the IL-4 time course for the indicated genes are shown, exhibiting: 1; reduced accessibility/expression (LOST), 2; gaining early accessibility/induction (EARLY) and showing late accessibility and late induction at the mRNA level (LATE). Small schematics indicate scATAC-seq data (Accessibility) and bulk RNA-seq data (mRNA level).

Supplementary Figure 2.

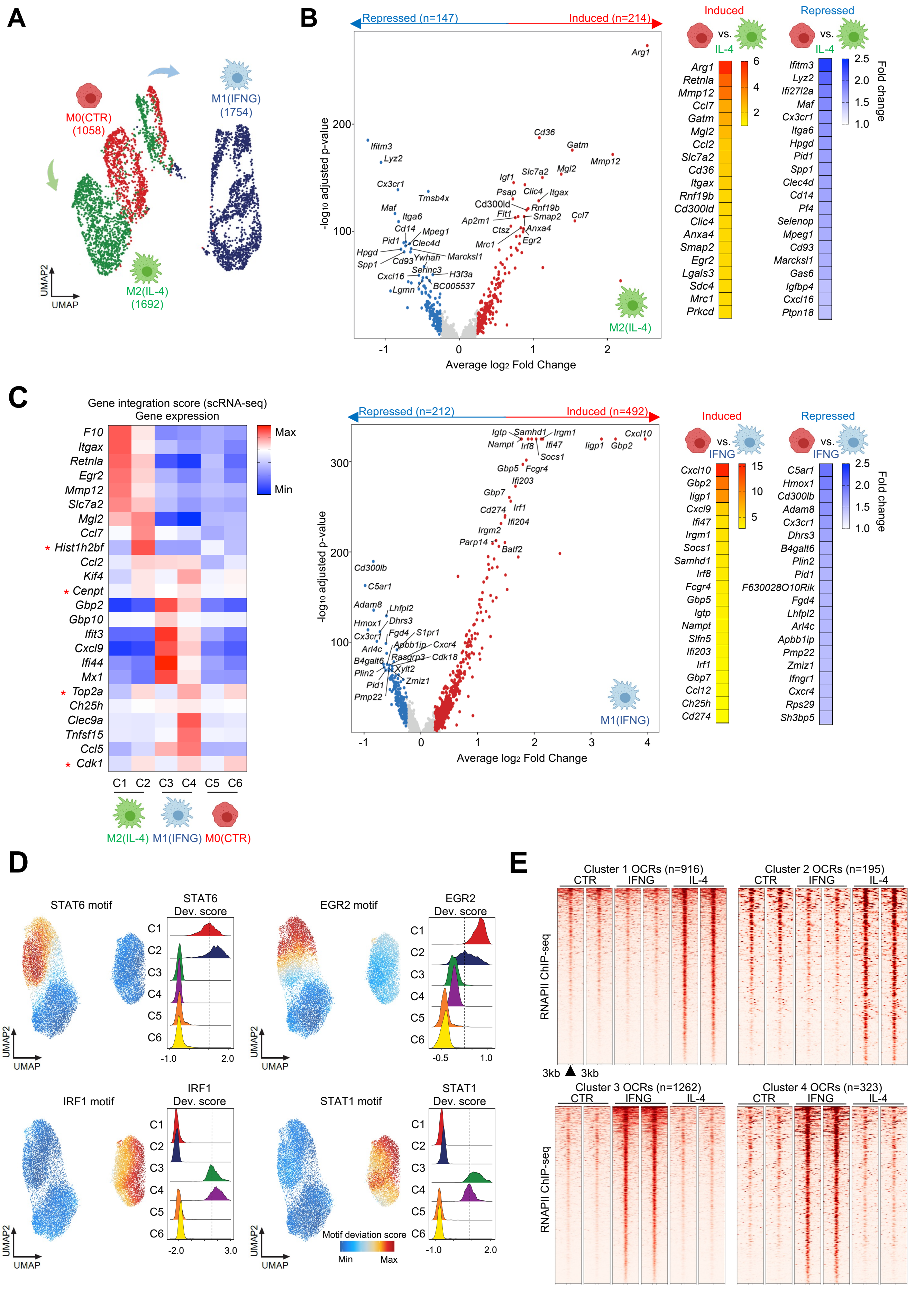

Figure S2. Single cell transcriptomic analysis of MF polarization. Related to Figure 2.

(A) UMAP of scRNA-seq experiments on M0(CTR), M2(IL-4) and M1(IFNG) macrophages. **(B)** Heatmap of differentially expressed genes in M0(CTR) vs. M2(IL-4) and M0(CTR) vs. M1(IFNG). Top20 induced and repressed genes are shown for both comparisons. Fold change over CTR is visualized. **(C)** Heatmap of integrated scRNA-seq expression values (Gene integration score), exhibiting biased expression level in the M2(IL-4) and M1(IFNG) scATAC-seq clusters. Red asterisks mark cell cycle genes. **(D)** ChromVAR transcription factor deviation scores (Dev.score) visualized either on the UMAP embeddings or as a ridge plot in the 6 clusters. **(E)** Read distribution plots of RNAPII ChIP-seq signals projected onto cluster specific open chromatin regions defined by scATAC-seq.

Supplementary Figure 3.

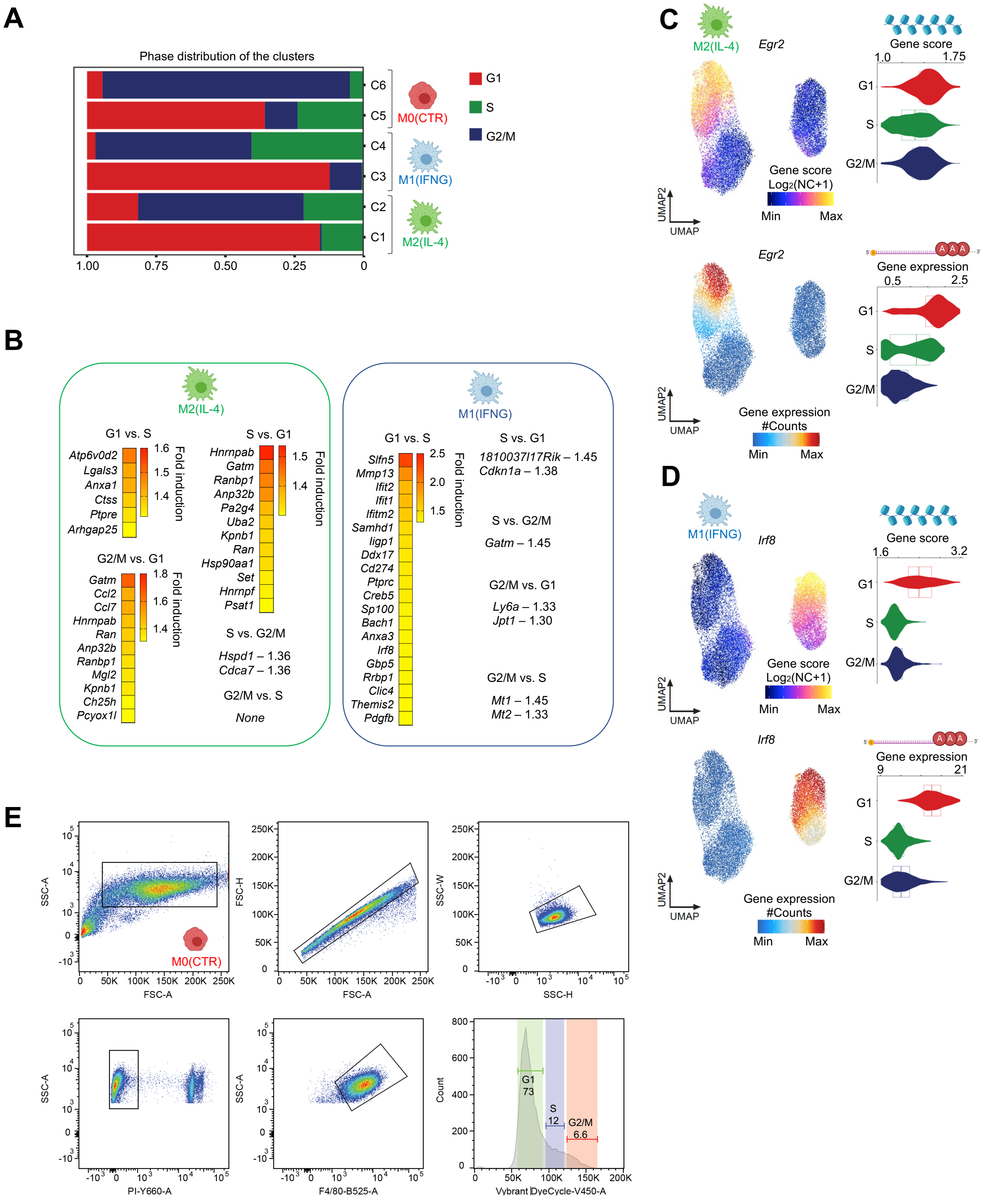

Figure S3. Prediction of cell cycle state in polarized MFs indicate reduced polarization ability in cell cycle. Related to Figure 3.
(A) Stacked bar plot depicts the distribution of macrophages across the 6 clusters as a function of their predicted cell cycle phase. **(B)** Predictions of cell cycle phase-biased gene expression using the integrated scRNA-seq values (gene integration matrix) projected onto the scATAC-seq clusters on panel Figure 3A. Comparisons across the different cell cycle phases are shown for both M2(IL-4) and M1(IFNG) exposed macrophages. **(C)** Gene scores and integrated gene expression values of *Egr2* (**(D)** for *Irf8*) are visualized on the UMAPs. Violin plots represent these values in the predicted cell cycle phases in either M2 (C) or M1 (D) polarized macrophages. **(E)** FACS gating strategy used to sort macrophages from distinct cell cycle phases.

Supplementary Figure 4.

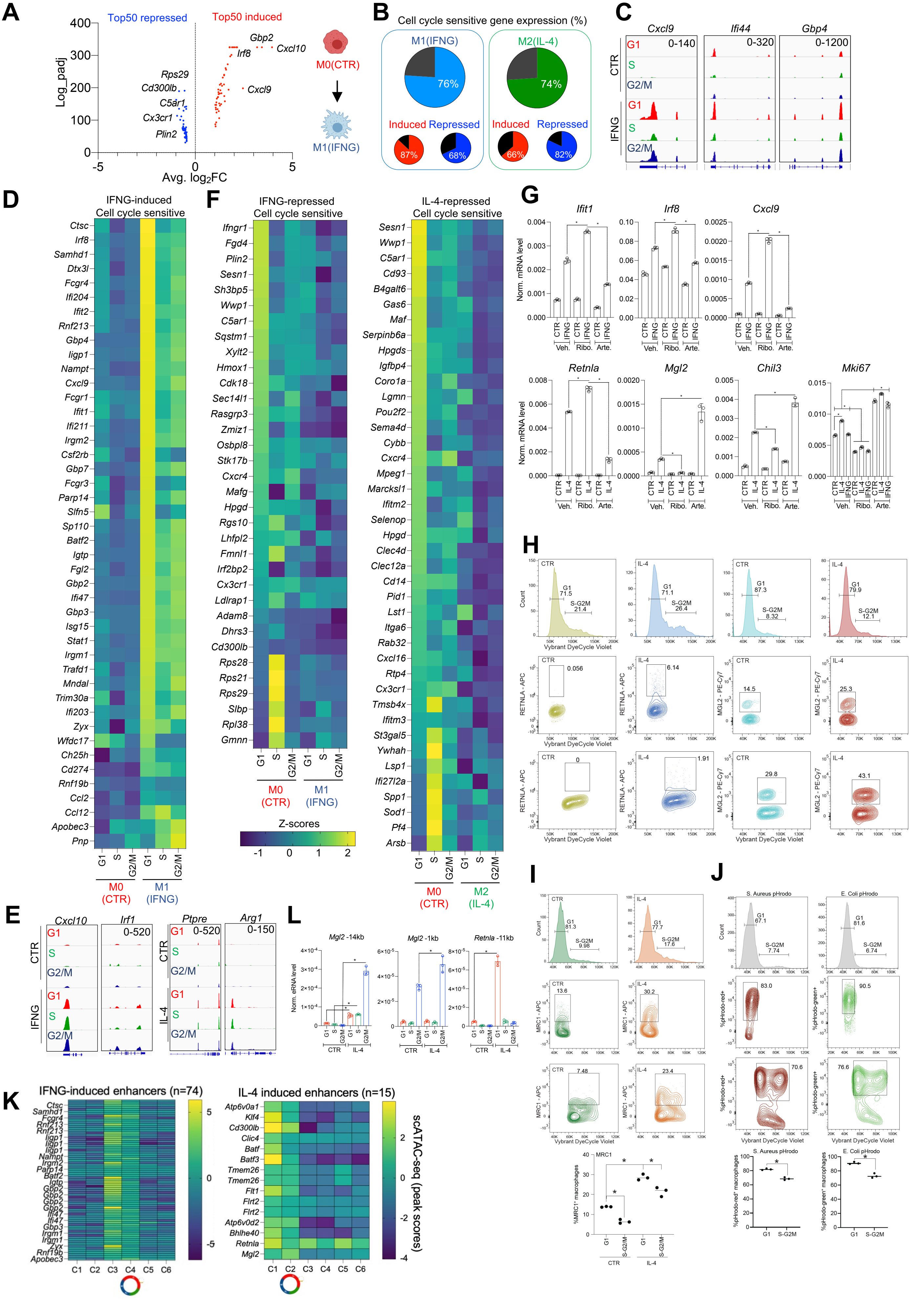

Figure S4. The transcriptional program of M1 and M2 polarized MFs in the different phases of cell cycle. Related to Figure 4.
(A) Volcano plot of top50 induced and repressed genes upon IFNG polarization as determined by scRNA-seq. **(B)** Pie charts depict the percentage of cell cycle sensitive gene expression in the two polarization models (top). Smaller pie charts show the percentage of cell cycle sensitive induced or repressed genes in the two polarization models. **(C)** Genome browser views of IFNG-induced genes with G1-biased expression. **(D)** Heatmap of IFNG-induced genes exhibiting cell cycle phase-biased expression. **(E)** Genome browser snapshots of genes with cell cycle-insensitive expression patterns in M1 and M2 macrophages, respectively. **(F)** Heatmaps of IFNG- and IL-4-repressed genes with cell cycle sensitive expression. **(G)** Gene expression measurements by RT-qPCR in the absence (Veh. – DMSO/EtOH), or in the presence of cell cycle inhibitors, Ribociclib (Ribo.) or Artesunate (Arte.). **(H)** Representative flow cytometry plots that depict the gating strategy to assess MGL2 and RETNLA positive fractions of macrophages in cell cycle, and validate cell cycle-biased expression at the protein level. Histograms show G1 and S-G2/M gates in which MGL2 and RENTLA positive macrophage fractions were quantified in untreated (CTR) and M2 (IL-4) polarized states. Quantification of this data is presented in Figure 4F. Quantification of the data is presented in Figure 4G. **(I)** Same as H for MRC1. **(J)** Representative flow cytometry plots that depict the gating strategy to assess the phagocytic activities of macrophages in cell cycle in the presence of the indicated bacterial particles. **(K)** Heatmaps represent the peak scores (accessibility) of enhancers with cell cycle-biased activities annotated to IFNG- and IL-4-induced genes with cell cycle-biased expression profiles. **(L)** Bar plots show eRNA levels of the indicated gene enhancers determined by RT-qPCR in the indicated conditions. In all bar plots and flow cytometry quantification plots, significant changes were determined by two tailed, unpaired t-test at $p<0.05$ ($n=3$). Shown are means with SDs.

Supplementary Figure 5.

A

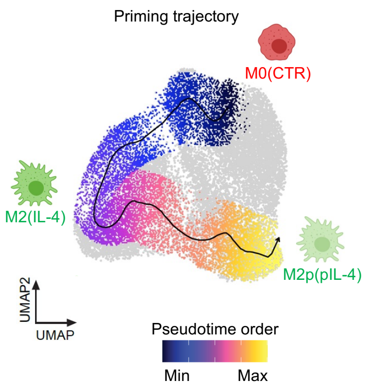

Figure S5. The chromatin state of IL-4-mediated MF priming. Related to Figure 5.
(A) UMAP of IL-4 priming trajectory (M0 – M2 – M2p).

Supplementary Figure 6.

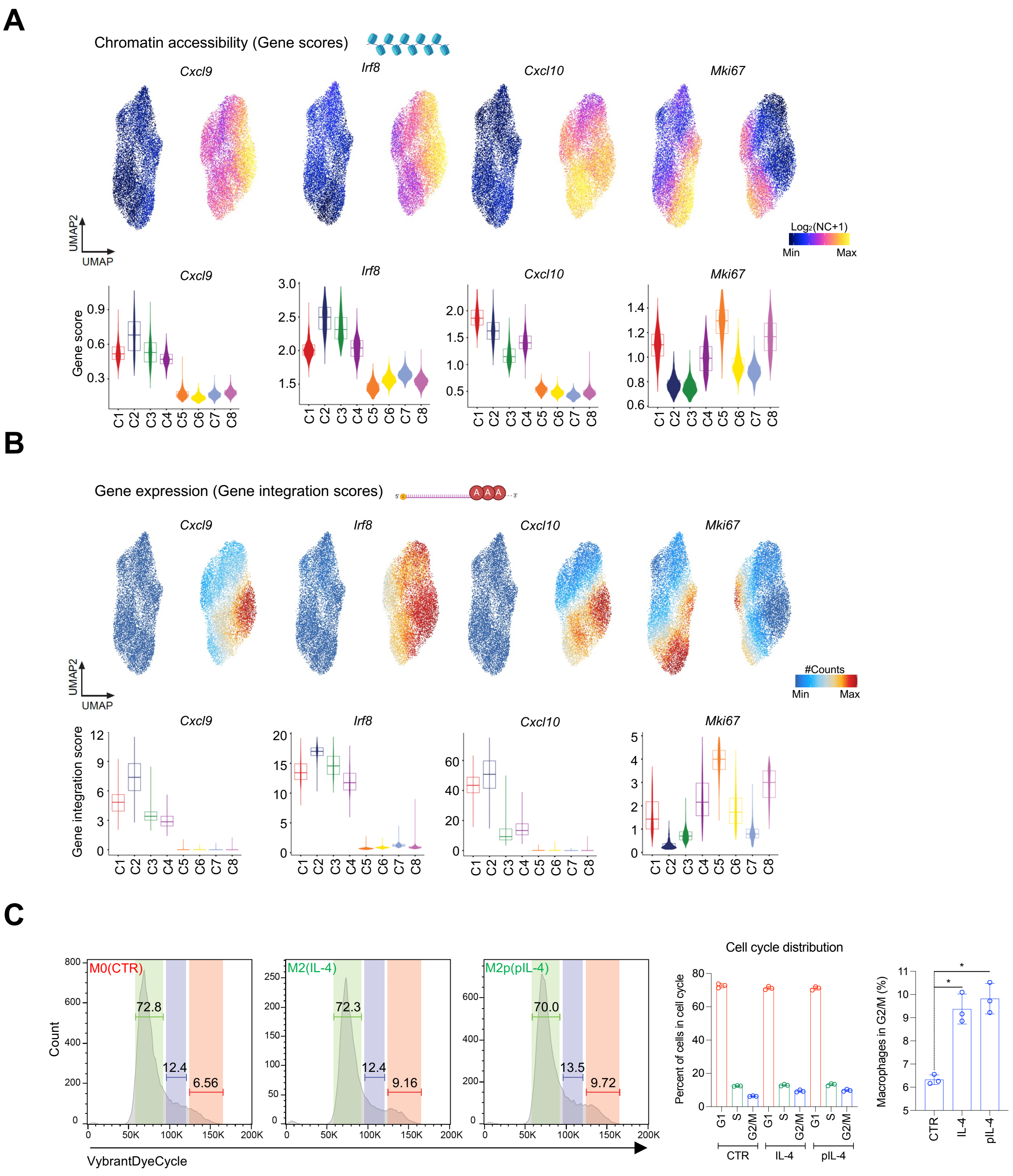

Figure S6. MF repolarization is negatively affected by cell cycle. Related to Figure 6.

(A) UMAPs and violin plots depict the gene score values (scATAC-seq) of the indicated genes. Log_2 normalized counts+1 is shown. **(B)** UMAPs and violin plots show the gene integration score values for the indicated genes. # - normalized **(C)** Representative FACS plot on the cell cycle phase distribution of M0, M2 and M2p macrophages (left). Percentage-wise cell cycle phase distribution of the samples (middle). Fraction of macrophages in the G2/M phase of cell cycle. Significant differences were identified by two tailed, unpaired t-test at $p<0.05$ (n=3) (right).

Supplementary Figure 7.

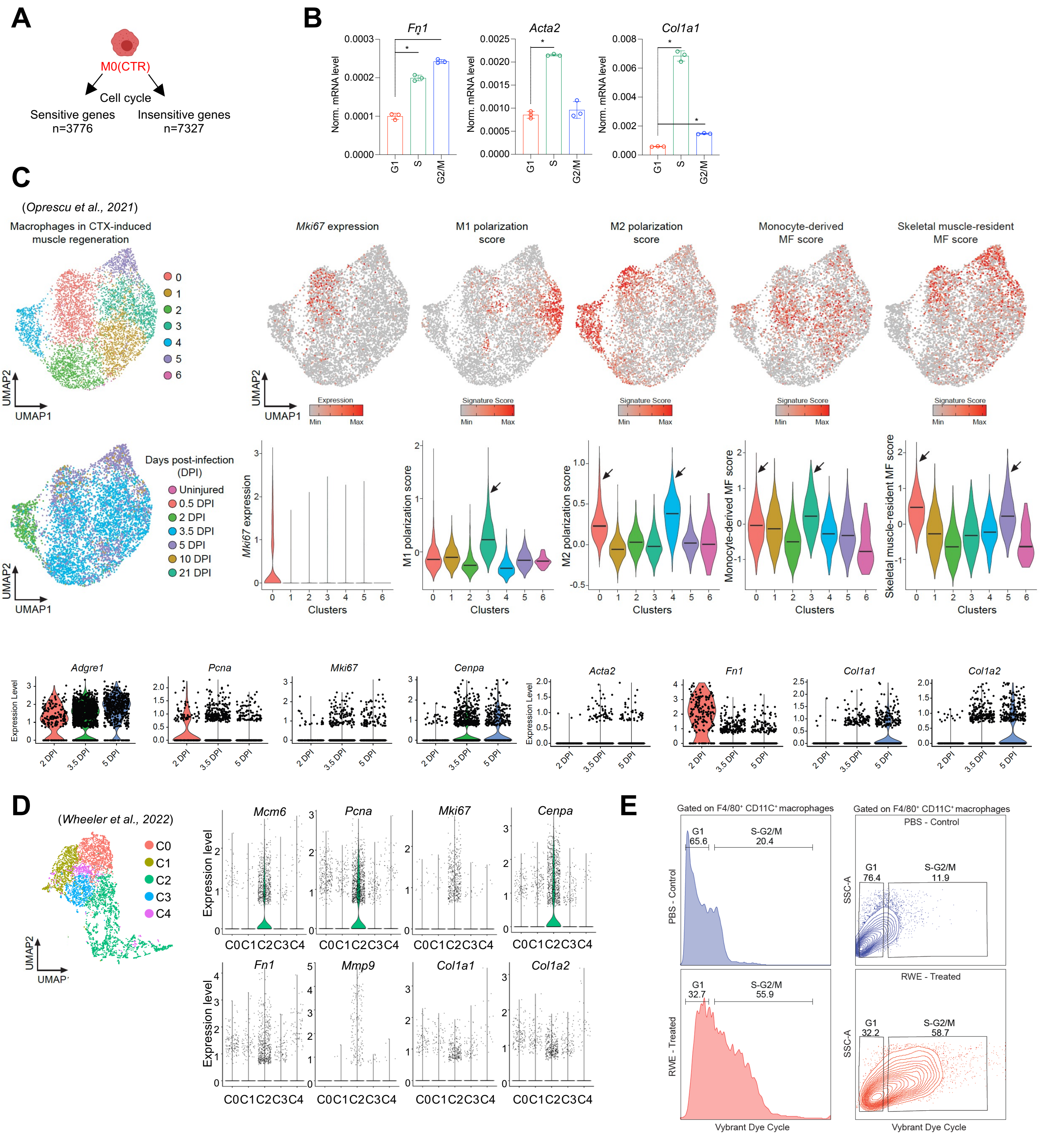

Figure S7. Proliferating MFs of regenerating tissues express tissue remodeling genes. Related to Figure 7.

(A) Scheme indicates the number of genes exhibiting cell cycle sensitive and insensitive expression patterns in M0(CTR) macrophages. **(B)** mRNA levels of *Fn1*, *Acta2* and *Col1a1* measured by RT-qPCR. Significant changes were determined by two tailed, unpaired t-test at $p<0.05$ (n=3). Shown are means with SDs. **(C)** scRNA-seq UMAP of macrophages colored by the clusters (left) or by the sample of origin (days post injury – DPI) (left bottom) (GSE138826). Individual UMAPs depict *Mki67* expression and the indicated gene signature scores. Violin plots quantify these features. Arrows point to clusters with the highest median gene signature scores. Violin plots show the expression of macrophage markers, cell cycle markers and tissue remodeling associated genes. **(D)** scRNA-seq UMAP of macrophages derived from barium chloride injured tibialis anterior colored by the identified clusters. Violin plots show the expression of cell cycle markers and tissue remodeling associated genes. **(E)** Gating strategy to sort alveolar macrophages from G1 and S-G2/M phases of cell cycle.
